# Ex vivo and in vivo fluorescence detection and imaging of adenosine triphosphate

**DOI:** 10.1186/s12951-021-00930-4

**Published:** 2021-06-22

**Authors:** Binbin Chu, Ajun Wang, Liang Cheng, Runzhi Chen, Huayi Shi, Bin Song, Fenglin Dong, Houyu Wang, Yao He

**Affiliations:** 1 The First Affiliated Hospital of Soochow University, Soochow University, Suzhou, 215006 Jiangsu China; 2 Laboratory of Nanoscale Biochemical Analysis, Jiangsu Key Laboratory for Carbon-Based Functional Materials and Devices, Institute of Functional Nano and Soft Materials (FUNSOM), Soochow University, Suzhou, 215123 China

**Keywords:** Nanoprobes, ATP, Titanium carbide, Detection, Fluorescence imaging

## Abstract

**Background:**

Ex vivo and in vivo detection and imaging of adenosine triphosphate (ATP) is critically important for the diagnosis and treatment of diseases, which still remains challenges up to present.

**Results:**

We herein demonstrate that ATP could be fluorescently detected and imaged ex vivo and in vivo. In particular, we fabricate a kind of fluorescent ATP probes, which are made of titanium carbide (TC) nanosheets modified with the ROX-tagged ATP-aptamer (TC/Apt). In the constructed TC/Apt, TC shows superior quenching efficiency against ROX (e.g., ~ 97%). While in the presence of ATP, ROX-tagged aptamer is released from TC surface, leading to the recovery of fluorescence of ROX under the 545-nm excitation. Consequently, a wide dynamic range from 1 μM to 1.5 mM ATP and a high sensitivity with a limit of detection (LOD) down to 0.2 μM ATP can be readily achieved by the prepared TC/Apt. We further demonstrate that the as-prepared TC/Apt probe is feasible for accurate discrimination of ATP in different samples including living cells, body fluids (e.g., mouse serum, mouse urine and human serum) and mouse tumor models.

**Conclusions:**

Fluorescence detection and imaging of ATP could be readily achieved in living cells, body fluids (e.g., urine and serum), as well as mouse tumor model through a new kind of fluorescent ATP nanoprobes, offering new powerful tools for the treatment of diseases related to abnormal fluctuation of ATP concentration.
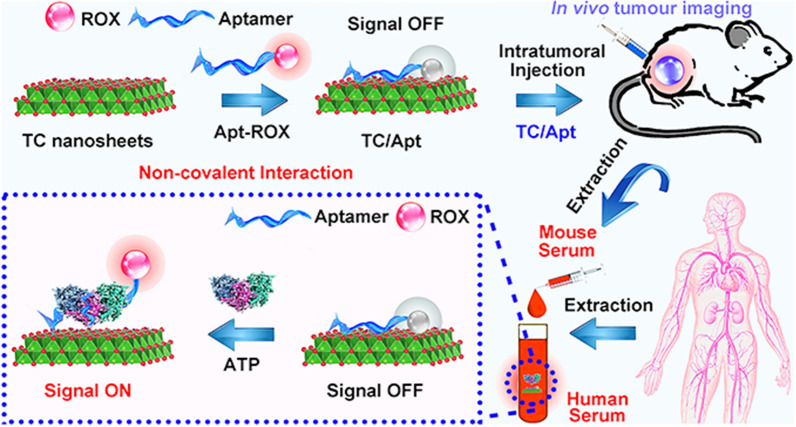

**Supplementary Information:**

The online version contains supplementary material available at 10.1186/s12951-021-00930-4.

## Introduction

Adenosine triphosphate (ATP), known as a primary energy storage molecule, plays a critical role in the regulation of cellular metabolism and biochemical pathways in a varied cell physiology [1, 2]. Several diseases are closely related to abnormal fluctuation of ATP concentration, such as angiocardiopathy, Parkinson’s disease, Alzheimer's disease, colitis, and malignant tumours [3–6]. For example, intracellular ATP contents in normal cells generally range from 1 to 10 mM; comparatively, the ATP concentration in cancer cells is significantly higher due to increasing glycolysis during tumour proliferation and angiogenesis [5–7]. Considering the significant role of ATP in these indicating diseases, it is essential for designing accurate and sensitive sensing strategies for imaging and sensing ATP, preferably satisfying the requirements of ex vivo and in vivo assays simultaneously.

To this end, numerous approaches have been developed for ATP detection, including surface-enhanced Raman scattering, electrochemistry, chemiluminescence, colorimetry, fluorescence and so on [8–16]. Among these methods, fluorescent probes for imaging and/or sensing intracellular ATP variations have attracted enormous attention due to their high sensitivity, good selectivity, convenient measurement and low cost [1, 17–20]. Most fluorescent probes for ATP detection were based on the fluorescence resonance energy transfer (FRET) sensing strategy. FRET is a nonradiative energy transfer process between donor chromophore and acceptor chromophore (quencher) [21–23]. When using the same donor in the FRET system, the acceptor featuring superior quenching efficiency would in principle lead to higher efficiency of the energy transfer. As such, searching for FRET acceptors with prominent quenching efficiency against fluorescent dyes is pivotal for the construction of ultrasensitive fluorescent ATP probes. To date, the quenching efficiency of most reported FRET acceptors were less than 95% [22, 23]. Besides, fluorescent probes suitable for simultaneously sensing ATP ex vivo and in vivo are still few.

**Fig. 1:**
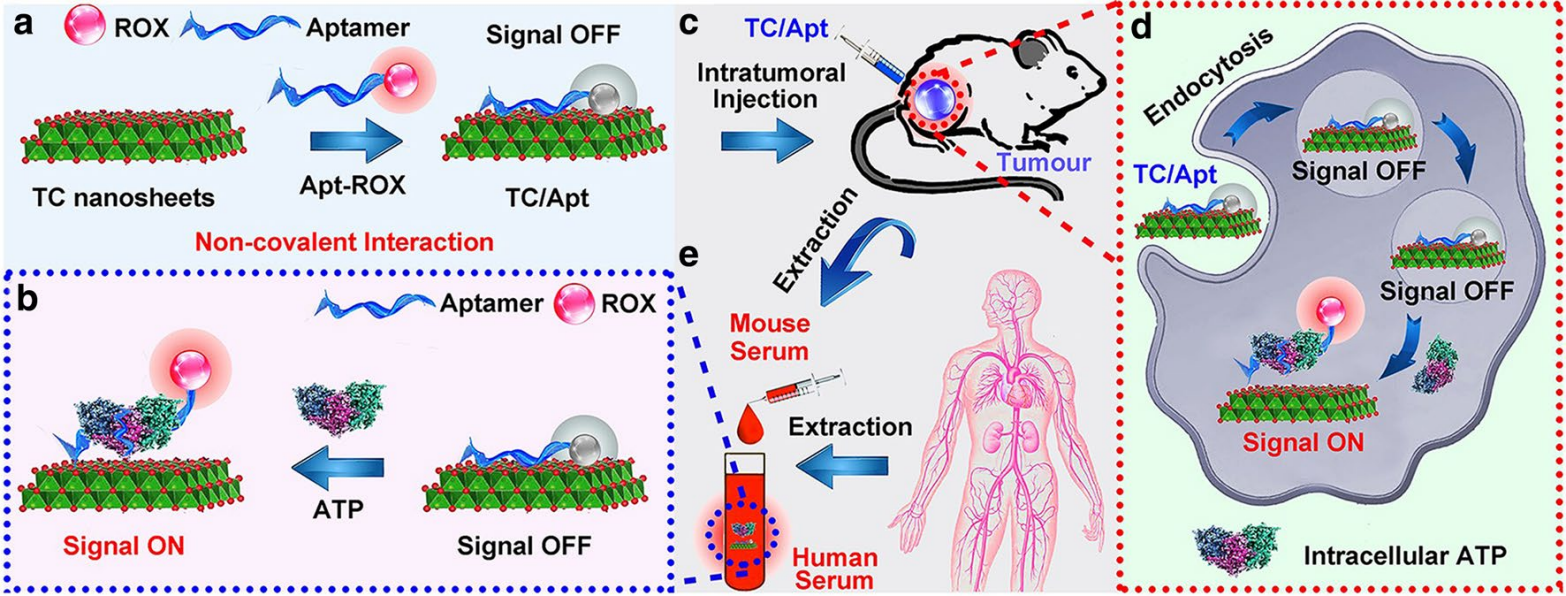
Scheme of TC/Apt fluorescent probes. **a** Schematic diagram of fabrication of TC/Apt-based fluorescent probes. **b** TC/Apt-based fluorescent probes for the accurate detection ATP based on signal off/on switch mechanism. **c** TC/Apt-based fluorescent probes for in vivo imaging of ATP in tumours of mouse model. **d** TC/Apt-based fluorescent probes for imaging analysis of intracellular ATP in live cells. **e** TC/Apt-based fluorescent probes for quantitative detection of ATP in real samples, such as mouse and human serum

Herein, we intend to present a kind of transition metal carbides and carbonitrides (MXenes)-based fluorescent probes, which is high-efficacy for in vitro and in vivo detection of ATP (as shown in Fig. 1). The probes are made of the two-dimensional titanium carbide (TC) nanosheets modified with the ROX-tagged ATP-aptamer (namely as TC/Apt), in which TC shows superior quenching efficiency against ROX (e.g., ~ 97%). The synthesized TC/Apt can be used for the sensitive detection of the intracellular ATP in cancer cells under the single 545-nm excitation. Such TC/Apt-based probes allow a broad linear range of 1 μM to 1.5 mM with a low limit of detection (LOD) down to 0.2 μM in the detection of ATP content. We further demonstrate that the as-prepared TC/Apt-based probes are capable for quantitatively detecting the content of ATP in mouse serum, mouse urine, human serum, as well as tumour tissues in living mouse.

## Materials and methods

### Synthesis of probes

The established MXenes were prepared via organic-base-driven intercalation and delamination method reported previously [24]. Firstly, the M_n+1_AX_n_ phase Ti_3_AlC_2_ nanosheets were etched by the HF aqueous solution to remove Al layer, and then TMAOH organic alkali solution was added to etch them and gotten TC nanosheets. Secondly, 1 mL mixed solution containing TC (200 μg/mL) and ROX-labelled ATP-aptamer (Apt-ROX, 500 nM) was transferred to a 1.5 mL centrifuge tube at room temperature, and then standing in the dark for 15 min. The aptamer sequence is listed as follows: ACCTGGGGGAGTATTGCGGAGGAAGGT-ROX. Then, these ATP-aptamer modified TC sheets (TC/Apt) were obtained via centrifugation at 12,000 rpm for 10 min. The unabsorbed aptamers were removed by the centrifugation (12,000 rpm, 10 min) for three times. Finally, the collected TC/Apt stored in the dark for the following experiments. The mass extinction coefficient of 29.1 Lg^−1^ cm^−1^ is used for the determination of TC/Apt concentrations [24].

### Imaging of intracellular ATP

Human cervical cells (HeLa cells), mouse 4T1 mammary carcinoma (4T1 cells), Human breast adenocarcinoma cells (MCF-7 cells), and Human retinal epithelial cells (ARPE-19 cells) were respectively incubated with 100 μg/mL of TC/Apt for 12 h at 37 °C. The excess TC/Apt was eliminated by using PBS buffer (pH 7.4) to rinse cells for several times. Next, the TC/Apt-treated cells were further incubated with 5 mM Ca^2+^ or 0.1 mM etoposide at 37 °C for another 2 h, respectively [18]. The live cell fluorescence imaging of ATP was performed by using a confocal laser scanning microscope (CLSM, Leica, TCS-SP5 II). To avoid cell damage caused by laser, the 30% power of diode laser was adopted. To reduce self-fluorescence interference of cell lines, the microscope offset was set as − 3%. The 560–650 nm channels were selected to collect TC/Apt probes fluorescence emissions under 543-nm excitation. The region of interest (ROI) in collected images was analyzed by image software (Leica LAS AF Lite). Of note, all images were collected under the same brightness and the same contrast.

### Detection of ATP in body fluids

The serum and urine samples were extracted from mouse or human. ATP solutions covering from 0.0 to 1.0 mM were separately spiked into the 0.1% diluted three different body fluid samples (e.g., mouse serum, mouse urine, and human serum), respectively [10]. Finally, the fluorescence intensity at 610 nm of the TC/Apt-based fluorescent probes could be detected and calculated from a series of PL spectra of ROX dyes after adding 200 μg/mL TC/Apt probes into above three different body fluid samples containing the ATP with various concentrations. Ethics approvals were obtained from the Ethics Committee of First Affiliated Hospital of Soochow University. Informed consent was achieved from all subjects before sample collection.

### Detection of ATP in vivo

These 4T1 or MCF-7 tumour-bearing mice were used as in vivo models in our experiments. For the in vivo imaging, the 0.1 mL cell suspensions of 4T1 (containing 5 × 10^6^ cells) or MCF-7 cells (containing 5 × 10^7^ cells) were subcutaneously injected into the right back region of Balb/c nude mice (female, 4–6 weeks old). After that, the PBS buffer were subcutaneously injected into the left back region of Balb/c nude mice. The mice whose tumour size was up to ~ 100 mm^3^ were randomly divided into two groups. Each group was intratumorally administered with TC/Apt probes with the concentration at 200 μg/mL (V = 0.1 mL). The TC/Apt probes-treated mice were euthanized after the 1-day period post-administration, and imaged by the Maestro EX in vivo fluorescence imaging systems (CRi, Inc.). All above animal experimental procedures were performed according to the Guideline for Animal Experimentation with the approval of the animal care committee of Soochow University.

### Statistical analysis

The confocal images were processed by the commercial image analysis software (Leica Application Suite Advanced Fluorescence Lite, LAS AF Lite) and common software of ImageJ (NIH Image; http://rsbweb.nih.gov/ij/). Error bars represent the standard deviation obtained from three independent measurements. All statistical analyses were performed using the Origin and GraphPad Prism 7 software. The statistical significance of differences was determined by a one-way ANOVA analysis. p < 0.05 (*), p < 0.01 (**) and p < 0.001 (***) were used to indicate statistical difference.

## Results and discussion

### Characterization of probes

**Fig. 2:**
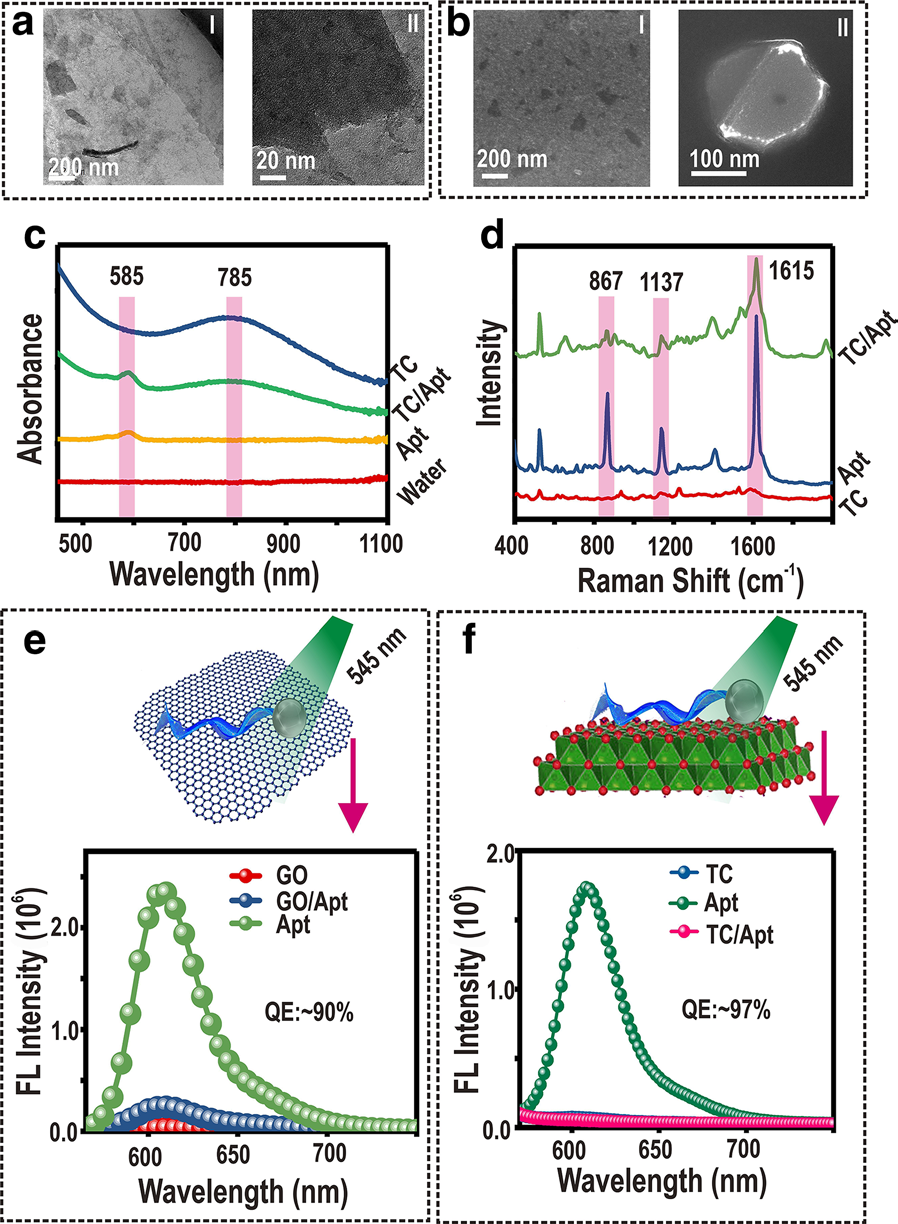
Characterizations of the as-prepared TC/Apt. **a** TEM (I) and HRTEM (II) images of TC/Apt. **b** SEM (I) and zoom-in SEM (II) images of TC/Apt. **c** Absorption spectra of deionized water (red line), free TC nanosheets (blue line), Apt (yellow line), and the TC/Apt (green line). **d** Raman spectra of free TC nanosheets (red line), Apt (blue line), and the TC/Apt (green line). **e** Photoluminescence (PL) spectra of free GO (red line), GO/Apt (blue line), and Apt (green line). **f** The PL spectra of free TC nanosheets (blue line), Apt (green line), and the TC/Apt (red line). Apt concentration: 500 nM, GO concentration: 200 μg/mL, and TC concentration: 200 μg/mL

The ROX-labelled ATP-aptamer (Apt-ROX) can be facilely and selectively adsorbed onto the prepared TC nanosheets by hydrogen bond and metal chelate interaction between the aptamer and TC nanosheets, guaranteeing the proximity of ROX to TC nanosheets surface (Fig. 1a). As thus, the fluorescence of ROX is rapidly and efficiently quenched by the TC nanosheets due to long-range energy transfer from ROX to these two-dimensional TC nanosheets (Fig. 1a). The as-prepared TC/Apt complex could be utilized for in vivo imaging ATP in tumour tissues of these mouse models based on signal off/on switch mechanism (Fig. 1b, c). Typically, the TC/Apt can burst into live cells based on active endocytosis, and then finally distribute in cellular cytoplasm (Fig. 1d). Then the hybridization between intracellular ATP and aptamer occurs due to the strong affinity between target and aptamer. Such hybridization leads to release of ROX-tagged aptamer from quencher surface, thus recovering the fluorescence of ROX under the 545-nm excitation (Fig. 1d). In addition to the in vivo imaging analysis, the resultant TC/Apt probes have the ability to quantitatively and sensitively detect ATP content in real samples, such as mouse and human serum (Fig. 1e). Firstly, surface topography and size distribution of as-prepared TC/Apt are imaged and analyzed by the transmission electron microscopy (TEM), high-resolution TEM (HRTEM), scanning electron microscopy (SEM) and dynamic light scattering (DLS), respectively. TEM and SEM images (Fig. 2a, b) reveal that the TC/Apt present obvious sheet-like structure with ~ 200–300 nm lateral dimensions coupled with the good dispersibility (Additional file 1: Figures S1a, 1b), which is consistent with pristine TC nanosheets (Additional file 1: Figures S1c–f). Furthermore, TC/Apt platforms are characterized by the photoluminescence (PL) spectroscopy, UV–Vis absorption spectroscopy, Raman spectroscopy, Fourier transform infrared (FTIR) spectroscopy, and Zeta potentials to validate successful conjugation of as-prepared TC nanosheets with ROX-tagged aptamer. Typically, as revealed in absorption spectra in Fig. 2c, pure Apt has a maximal peak at ~ 585 nm (yellow line) assigned to ROX moiety [25], and pure TC nanosheets have a strong absorption at ~ 785 nm attributed to the modified Al oxoanions [24]. As a result, TC/Apt complex shows two typical peaks at ~ 585 nm and ~ 785 nm. Moreover, Fig. 2d provides Raman spectra of TC nanosheets, Apt, and TC/Apt. In particular, three characteristic Raman peaks at 867, 1137, and 1615 cm^−1^ are observed in Apt, assigned to ν_(C–C)_ ring-stretch of ROX [26, 27]. These Raman bands can be observed in TC/Apt complex rather than in pure TC nanosheets.

In order to evaluate the quenching efficiency (QE) of TC nanosheets against ROX, graphene oxide (GO) with the same concentration of 200 μg/mL is selected for a comparison. Typically, GO as well as MXenes belong to 2D nanosheets, featuring similar physical/chemical properties such as electron confinement in ultrathin two dimensions, large cargo loadings, high aspect ratios and so forth [28]. Of note, GO-based probes been developed for imaging ATP in living cells, in which GO also serve as the FRET acceptors with high quenching efficiency against fluorescent dyes [17–19]. Taken together, herein we choose the established and representative GO as the control group to highlight the superior quenching efficiency of TC in the developed ATP probes. As shown in PL spectra in Fig. 2e, 500 nM Apt presents a typical emission peak at 610 nm, which sharply decreases when it is absorbed by GO to form GO/Apt complex. Typically, the PL intensity of pure Apt at 610 nm is ~ 9.8-fold stronger than that of GO/Apt complex, and the corresponding QE is calculated to be ~ 90% [18]. On the contrary, the stronger fluorescence quenching capacity of TC nanosheets against ROX is observed in Fig. 2f. The PL spectrum of TC/Apt complex is in line with pure TC nanosheets, and the corresponding QE is calculated to be ~ 97%. Typically, mass extinction coefficients of TC at 808 nm are calculated to be 29.1 Lg^−1^ cm^−1^ based on the Lambert–Beer law, which is much higher than that of 3.6 Lg^−1^ cm^−1^ for GO and 24.6 Lg^−1^ cm^−1^ for the reduced GO [24]. Secondly, compared with GO, the resultant TC nanosheets feature much more forceful light absorption from visible to near infrared (NIR) region [28–31]. Thereby, the fluorescence quenching capacity of TC nanosheets against ROX is stronger than that of GO in this developed system. Meanwhile, fluorescence quenching ability of TC nanosheets would not be influenced by both different temperatures and pH values, suggesting the relatively stable structure of as-prepared TC/Apt platforms in various environments (Additional file 1: Figures S2–3). Furthermore, the hydrogen bond of the as-prepared TC/Apt are characterized by FTIR and fluorescence intensity changes in different environments (Additional file 1: Figure S4). In brief, the peak of aptamer including O–H at ~ 1383 cm^−1^, which is covered by the TC nanosheets; and the TC/Apt probes show a shoulder peak of the carbonyl (C=O) stretching band at ~ 1640 cm^−1^ with a slight peak shift to short wavenumber (Additional file 1: Figures S4a–c) [32]. In addition, a high concentration of urea can destroy the hydrogen bond, which is chosen for confirming the existence of a hydrogen bond [33]. The fluorescence recovery slightly increases after adding urea (p < 0.001), also indicating the existence of a hydrogen bond between TC nanosheets and Apt-ROX (Additional file 1: Figures S4a–d) [33]. Taken together, these results demonstrate that The Apt-ROX can be facilely and selectively adsorbed onto TC nanosheets by hydrogen bond between aptamer and TC [32, 33]. With regard to Zeta potentials (Additional file 1: Figure S5), the zeta potential decreases from higher negative charge (ca. – 33.4 mV) to lower negative charge (ca. −16.4 mV) when Apt links to TC.

### Quantitative determination of ATP

**Fig. 3:**
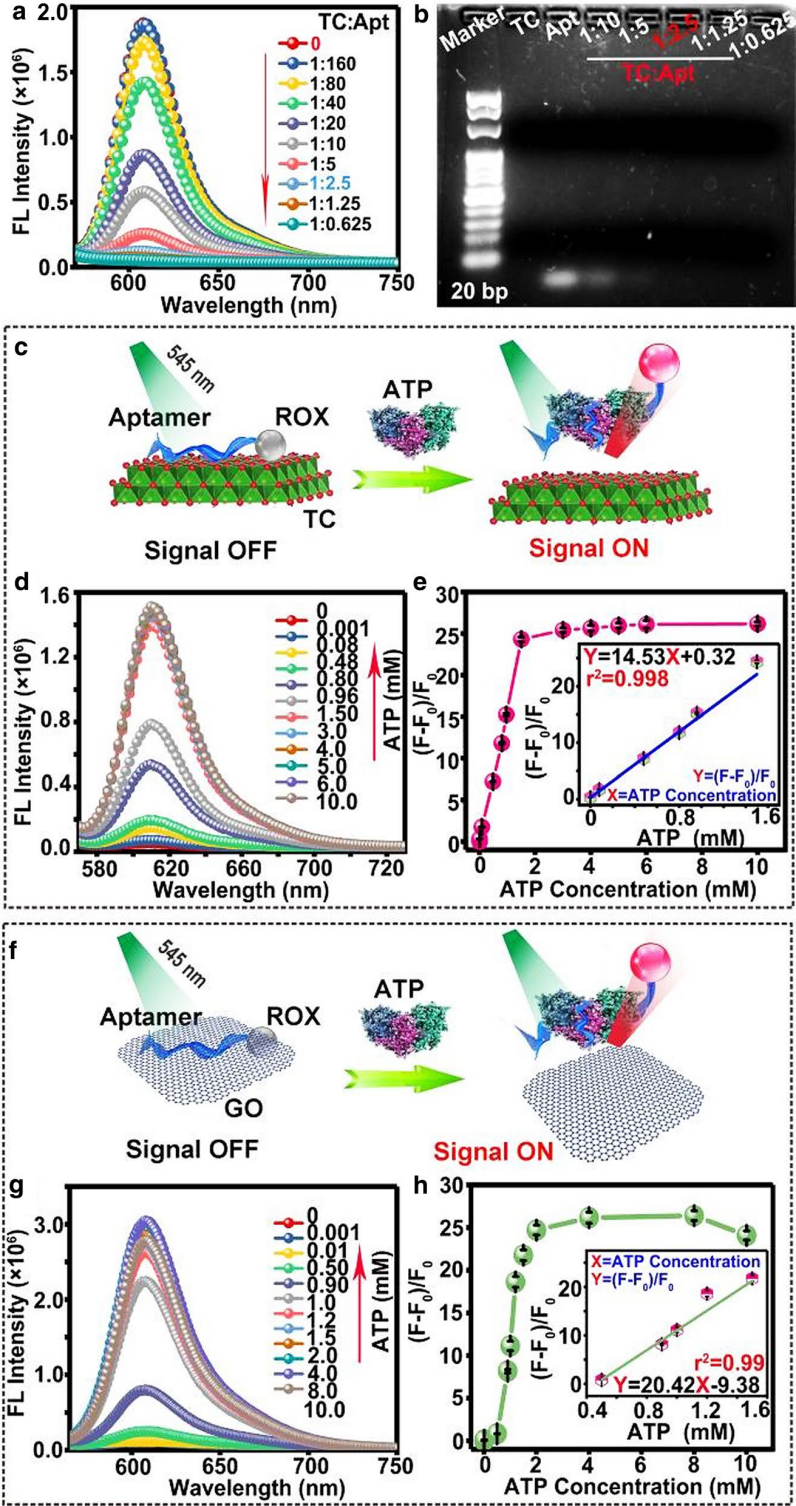
Quantitative determination of ATP. **a** PL spectrum of the TC/Apt made of different concentration ratios of TC nanosheets to Apt under the 545-nm excitation. **b** Corresponding agarose gel electropherogram of TC, Apt, and TC/Apt synthesized with different concentration ratios. **c–e** Schematic diagram of TC/Apt for biosensing ATP under 545 nm excitation (**c**). PL spectra of TC/Apt treated with ATP at various concentrations (**d**), and corresponding scatter diagrams of the relative fluorescence intensity ratio ((F-F_0_)/F_0_) versus the ATP concentration (**e**). **f–h** Schematic diagram of GO/Apt for the ATP biosensing under the 545 nm excitation (**f**). PL spectra of GO/Apt treated with ATP at various concentrations (**g**), and corresponding scatter diagram of relative fluorescence intensity ratio ((F − F_0_)/F_0_) versus ATP concentration (**h**). The insets represent the calibration curve of ((F − F_0)_/F_0_) versus ATP concentration. TC/Apt and GO/Apt: 200 μg/mL. ATP: 0.001–10 mM

The concentration ratio of TC nanosheets to Apt is a vital factor for fluorescence quenching effect of TC/Apt platforms. Figure 3a displays PL spectra of TC/Apt complex, produced in different concentration ratios of TC nanosheets to Apt (i.e., 1:0.625–1:160). Particularly, the weakest fluorescence intensity of TC/Apt is observed when the concentration ratio equals to or more than 1:2.5. As shown in the corresponding agarose gel electropherogram of Fig. 3b, no free aptamer bands in supernatant can be observed when the concentration ratio of TC nanosheets (200 μg/mL) to Apt (500 nM) is above 1:2.5, also confirming the ratio of 1: 2.5 is the optimal concentration ratio to prepare TC/Apt. Thus, the final concentration of TC nanosheets and Apt-ROX is 200 μg/mL and 500 nM, respectively, in this system of TC/Apt probes. Meanwhile, the thermogravimetric analysis (Additional file 1: Figure S6) also reveals that the Apt-ROX can be incorporated with TC nanosheets to construct TC/Apt probes and the mass ratio of Apt-ROX in TC/Apt can be calculated as ~ 1.8%. Before the detection of ATP in vitro, we firstly test the binding ability of ATP to lead Apt-ROX desorb from TC nanosheets. As shown in the agarose gel electrophoresis analysis (Additional file 1: Figure S7), the free ATP are able to desorb Apt-ROX from TC/Apt nanoprobes by specifically targeting the Apt-ROX and changing conformations of Apt-ROX. Figure 3c, f, respectively show schematic illustration of TC/Apt and GO/Apt for fluorescent detection of ATP. Figure 3d gives a series of PL spectra of TC/Apt under the 545-nm excitation when adding ATP solutions with different concentrations (e.g., 0.001–10.0 mM). Specifically, the fluorescence intensity of TC/Apt gradually grows with increase of ATP concentrations, revealing more Apt chains are released from TC sheets. The ATP concentration-dependent fluorescence change is further quantitatively explored in scatter curve of normalized fluorescence intensity ((F − F_0_)/F_0_) at the 610 nm versus ATP concentration (Fig. 3e). Herein, F or F_0_ represent the fluorescence intensity of TC/Apt treated with (F) or without ATP (F_0_), respectively. As revealed, the whole process from signal-off to signal-on state shows an ATP concentration-dependent manner. Notably, when the ATP concentration reaches 1.5 mM, the maximal (F − F_0_)/F_0_ value is achieved. While when ATP concentration further increases, no further significant enhancement is observed, indicating no more Apt is dissociated from TC nanosheets triggered by ATP. In addition, a good linearity is presented between the relative (F − F_0_)/F_0_ and ATP concentration in the range of 1 μΜ to 1.5 mM (Fig. 3e, inset). The corresponding regression equation is Y = 14.53 X + 0.32 with a good correlation coefficient of r^2^ = 0.998, where, Y represents (F − F_0_)/F_0_ value and X means ATP concentration. The corresponding limit of detection (LOD) is calculated to be ~ 0.2 μΜ by setting the signal-to-noise ratio of 3:1 (Fig. 3e, inset). Comparatively, the dynamic range of GO/Apt is from 0.5 mM to 1.5 mM under the same conditions, much narrower than that of TC/Apt (e.g., 1 μM–1.5 mM), as shown in Fig. 3g. Accordingly, the LOD of GO/Apt is down to 0.46 mM by setting the signal-to-noise ratio of 3:1 (Fig. 3h), which is two orders of magnitude higher than that of TC/Apt (e.g., 0.2 μΜ). The superior dynamic range and sensitivity of TC/Apt over GO/Apt is partially due to their higher quenching efficiency of the TC over GO (e.g., ~ 97% versus ~ 90%), as mentioned above (Additional file 1: Table S1) [24, 28–31]. Afterwards, the selectivity of TC/Apt-based platform for detection of ATP molecules is evaluated against other similar molecules such as uridine triphosphate (UTP), cytidine triphosphate (CTP), guanosine triphosphate (GTP), and adenosine monophosphate (AMP) (Additional file 1: Figure S8), different ions (Additional file 1: Figure S9), as well as various amino acids (Additional file 1: Figure S10). As depicted in Additional file 1: Figures S8–S10, those interfering species display much weak fluorescence response, whereas the ATP sample exhibits relatively strong fluorescence response, verifying the good selectivity of prepared TC/Apt-based probes.

### Detection of ATP in vitro

**Fig. 4:**
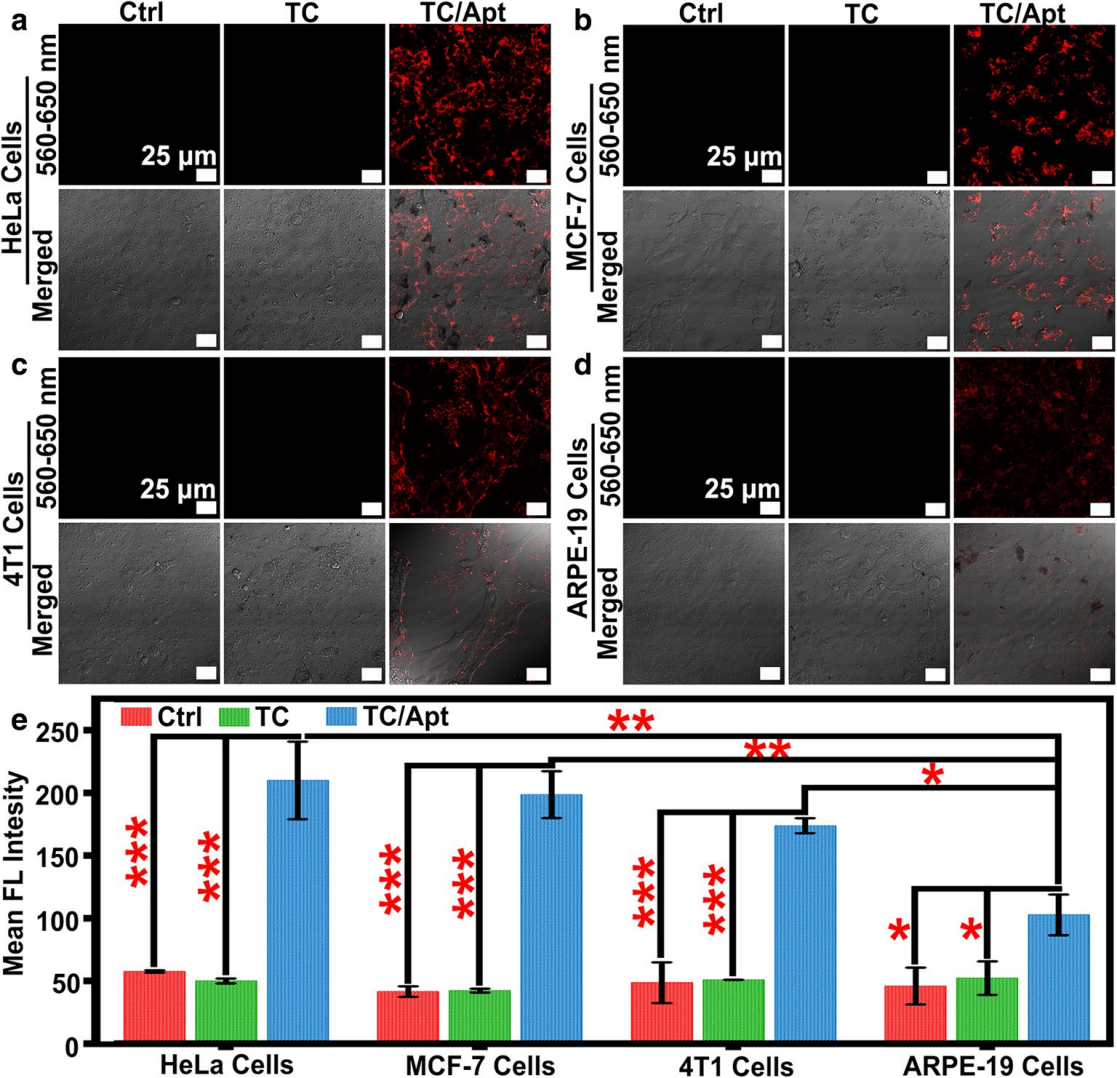
Intracellular imaging and sensing of ATP assisted by the TC/Apt. **a–d** CLSM images of TC/Apt in HeLa cells (**a**), MCF-7 (**b**), 4T1 (**c**), and ARPE-19 (**d**) cells. The TC/Apt-treated cells were respectively incubated with pure medium (control groups), free TC (100 μg/mL), and TC/Apt (100 μg/mL) at 37 °C for 12 h. Scale bars, 25 μm. **e** Corresponding histograms of the mean fluorescence intensity in HeLa cells, MCF-7 cells, 4T1, and ARPE-19 cells incubated with pure medium, pure TC, and the as-prepared TC/Apt probes, respectively. * represents p < 0.05, ** represents p < 0.01, and *** represents p < 0.001

**Fig. 5:**
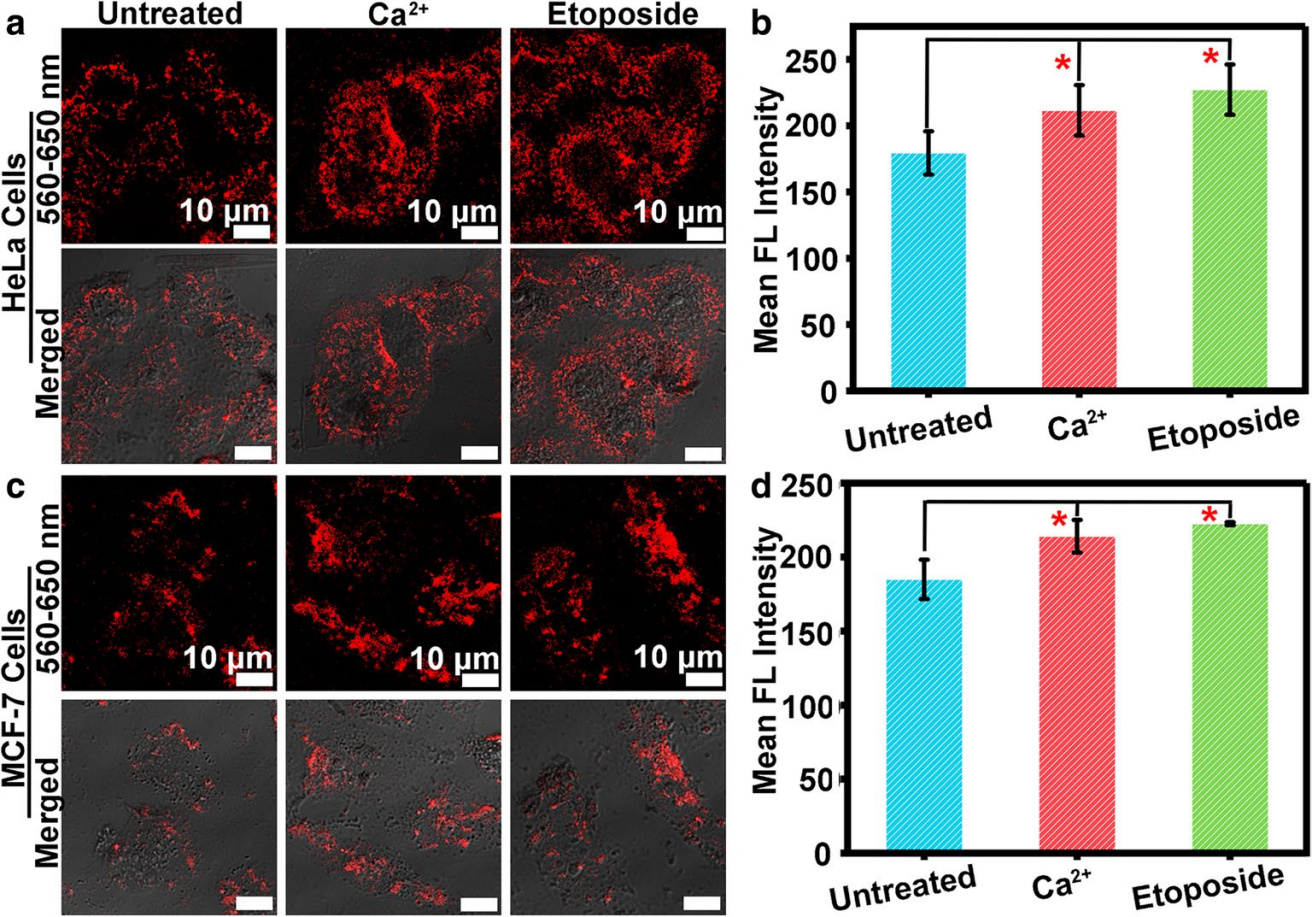
Imaging of intracellular ATP in living cells treated by Ca^2+^ (5 mM) or etoposide (0.1 mM). CLSM images of the resultant TC/Apt in HeLa cells (**a**) and MCF-7 cells (**c**) treated by the Ca^2+^ or etoposide for another 30 min. Scale bars, 10 μm. Corresponding histograms of the mean fluorescence intensity of the TC/Apt in HeLa cells (**b**) and MCF-7 cells (**d**). * represents p < 0.05

Next, we primarily evaluate the cytotoxicity of TC/Apt probes. The corresponding viabilities of treated cells are above ~ 85% (Additional file 1: Figure S11), suggesting low cytotoxicity of the resultant TC/Apt. Furthermore, the TC/Apt entering into cells is through the energy-consuming endocytosis [34, 35], as revealed in Additional file 1: Figures S12–15. Based on above results, the feasibility of these TC/Apt-based probes for sensing of intracellular ATP in cytoplasm is systematically evaluated. Experimentally, the unmodified TC (100 μg/mL) and TC/Apt probes (100 μg/mL) are respectively incubated with cancer cells (e.g., HeLa, MCF-7, and 4T1 cells) and normal cells (e.g., ARPE-19 cells) at 37 °C for 12 h, followed by the CLSM imaging. As shown in confocal images of the Fig. 4a–d, no fluorescence can be measured in cells without any treatments (control groups, Ctrl). Similarly, TC-treated cells also show negligible fluorescence signals. On the contrary, distinct red fluorescence can be observed in TC/Apt-treated HeLa (Fig. 4a), MCF-7 (Fig. 4b), and 4T1 cells (Fig. 4c). Comparatively, the relatively weak red fluorescence can be detected in these TC/Apt-treated ARPE-19 cells (Fig. 4d). These experimental results are consistent with previously reported results that ATP contents in cancer cells are much higher than those in normal cells [5–7]. For further quantitative comparison, the fluorescence intensity for each image is analyzed by using the commercial image software (Leica LAS AF Lite). The mean fluorescence intensity of Ctrl, TC, and TC/Apt groups is displayed in Fig. 4e. The mean fluorescence intensity of these TC/Apt-treated cancer cells is significantly higher than control groups (e.g., ~ 3.6–4.8 folds, p < 0.001) or TC groups (e.g., ~ 3.5–4.7 folds, p < 0.001), which is consistent with cellular imaging results mentioned above. Also, Fig. 4e further exhibits that the mean fluorescence intensities of cancer cells determined by TC/Apt probes are significantly higher than those of normal cells [e.g., ~ 1.7 fold of 4T1 cells (p < 0.05), ~ 1.9 fold of MCF-7 cells (p < 0.01), and ~ 2.0 fold of HeLa cells (p < 0.01)], which are in accordance with above cellular fluorescence images. These results demonstrate that TC/Apt probes are available for in situ monitoring of intracellular ATP in living cells. To demonstrate the capability of the TC/Apt to monitor ATP level changes inside living cells, the calcium ion (Ca^2+^) and etoposide are used to promote the ATP production of the cancer cells. In previous reports, Ca^2+^ and etoposide as apoptotic stimuli are widely employed for the elevation of cytosolic ATP level [18, 36]. For both HeLa and MCF-7 cells, in comparison to relatively weak fluorescence of cells without incubation with Ca^2+^ or etoposide (untreated groups), relatively strong fluorescence is measured in cells incubated with Ca^2+^ or etoposide (Fig. 5a, c). For the quantitative evaluation, mean fluorescence intensities of untreated, Ca^2+^ and etoposide groups are given in Fig. 5b, d. Compared with untreated cells (control groups), the mean fluorescence intensity increases by ~ 18% in Ca^2+^-treated HeLa cells, by ~ 20% in etoposide-treated HeLa cells (Fig. 5b), by ~ 16% in Ca^2+^-treated MCF-7 cells and by ~ 21% in these etoposide-treated MCF-7 cells (Fig. 5d). The similar results are observed in 4T1 cells (Additional file 1: Figure S16). These data indicate that TC/Apt probes have the ability to monitor level changes of intracellular ATP.

### Detection and imaging of ATP in body fluid

**Fig. 6:**
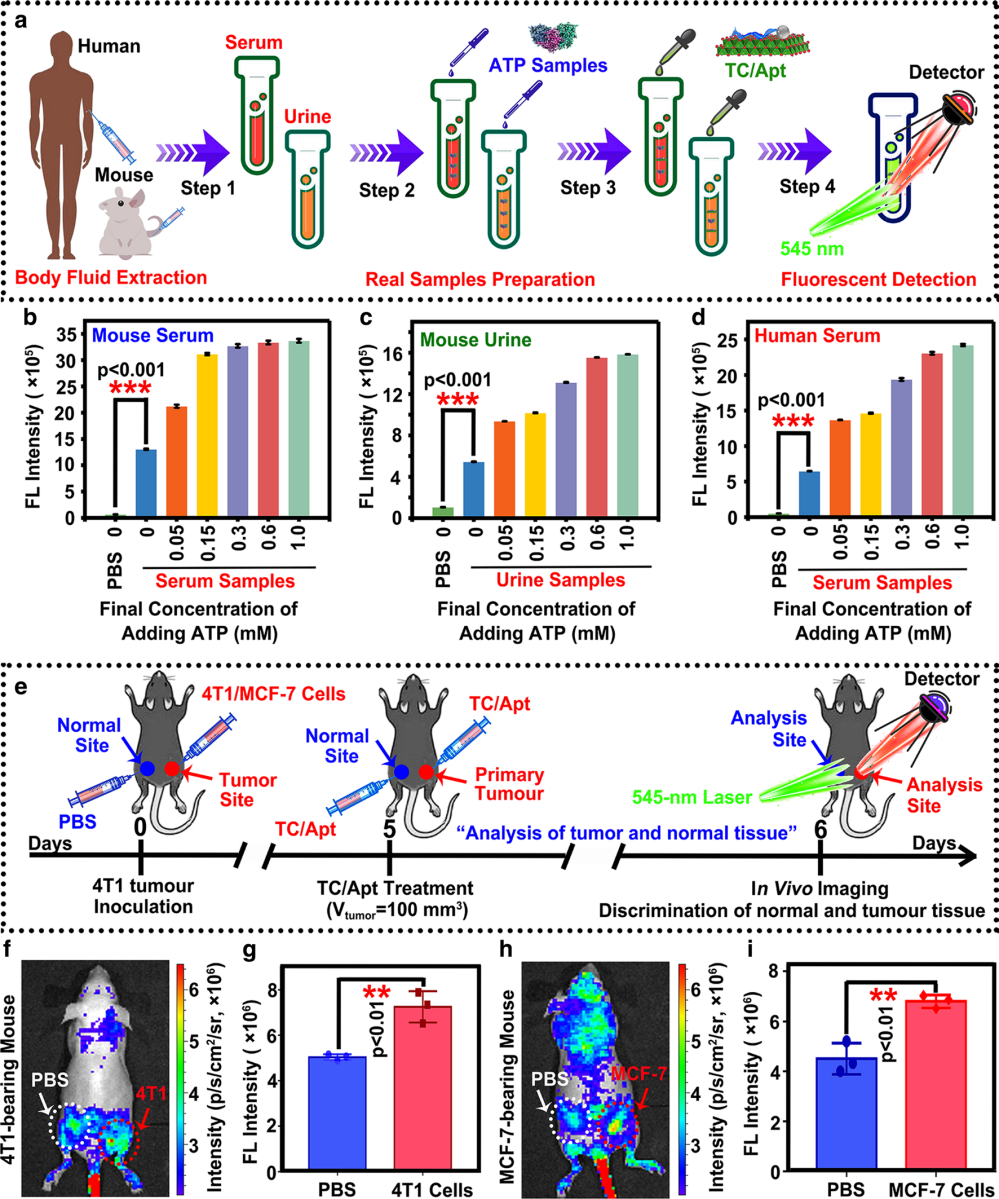
Imaging and sensing ATP in real complex samples. **a** Schematic diagram of TC/Apt fluorescent probes for detecting ATP concentration in real complex samples, such as mouse serum, mouse urine, and human serum. **b–d** Corresponding histogram of fluorescence intensity of TC/Apt probes in PBS, mouse serum (**b**), mouse urine (**c**) and human serum (**d**) with various final concentration of the adding ATP. *** means p < 0.001. **e** Schematic illustrating TC/Apt probes for in vivo imaging and monitoring of tumour tissues in a mouse model. **f** In vivo imaging of normal site and 4T1 tumor tissues after treatment with 100 μL TC/Apt of a concentration at 200 μg/mL for 6 h. **g** Corresponding histograms of fluorescence intensity on the left and right back of the 4T1 tumor-bearing mice. ** means p < 0.01. **h** In vivo imaging of normal site and MCF-7 tumor tissues after treatment with TC/Apt of a concentration at 200 μg/mL for 6 h. **i** Corresponding histograms of fluorescence intensity on the left and right back of the MCF-7 tumor-bearing mice. ** means p < 0.01

To evaluate the feasibility of the developed probes for imaging and sensing ATP in real complex samples, the fluorescent TC/Apt probes are utilized to detecting ATP with various concentrations in the different body fluid (Fig. 6a). In detail, serum and urine samples are extracted from mouse or human (Step 1). After that, series of concentrations of standard ATP covering from 0.0 to 1.0 mM are separately spiked into the 0.1% diluted different body fluid samples, respectively (Step 2) [10]. Finally, the fluorescence intensity at the 610 nm can be detected and calculated from a series of PL spectra of ROX dyes after adding the 200 μg/mL TC/Apt probes into above body fluid samples containing ATP with various concentrations (Steps 3–4). As depicted in Fig. 6b–d, the fluorescence intensity of the resultant TC/Apt probes in the original body fluid (i.e., spiked ATP concentration is 0 mM) is much higher than that in PBS groups (p < 0.001), indicating the existence of ATP in the original body fluids. In detail, when the spiked ATP concentration in mouse serum samples is 0 mM, the fluorescence intensity at 610 nm of the resultant TC/Apt probes can be detected as ~ 13 × 10^5^, which are much higher than ~ 6 × 10^4^ of PBS groups at the same content of the adding ATP (p < 0.001) [10]. With the increase of the spiked ATP concentration, fluorescence intensity at the 610 nm of TC/Apt probes in real complex samples gradually increases from ~ 13 × 10^5^ to ~ 34 × 10^5^, indicating that the prepared TC/Apt probes offer the ability to detect the ATP content in different body fluid samples. As further shown in Fig. 6c–d, mouse and urine human serum samples also exhibit similar experimental results and growth trend of the fluorescence intensity at 610 nm versus spiked ATP concentration. Significantly, as shown in mouse urine samples (Fig. 6c), the existence of urea can influence the fluorescence intensity of the prepared TC/Apt probes, [32, 33] the resultant TC/Apt cannot accurately analyzing the ATP content in urine samples. Taken together, these results demonstrate that the as-prepared TC/Apt probes can be used to detect the ATP content in real complex samples (e.g., serum, etc*.*). Furthermore, the 4T1 or MCF-7 tumor-bearing mouse model is employed for verify the feasibility of TC/Apt probes for imaging ATP in vivo (Fig. 6e). In detail, Fig. 6f displays that the TC/Apt-based fluorescent probes can exhibit more obvious fluorescence signals on the right back tumor site of the 4T1 tumor-bearing mouse after intratumoral injection with 200 μg/mL TC/Apt than that of left PBS-treated sites. In particular, the weak fluorescence signals observed in normal sites (e.g., brain, feet, etc*.*) belong to autofluorescence of mice, which cannot obviously affect the analysis and discussion of in vivo imaging analysis. However, high-performance probes (e.g., near-infrared or bioluminescent nanoprobes, etc*.*) need to be designed and developed for the higher-quality imaging and diagnosis of different diseases in clinical researches [37–39]. For further quantitative comparisons, the mean fluorescence intensity detected in the 4T1 tumor tissue is significantly higher than that of these PBS-treated skin tissues (p < 0.01) (Fig. 6g). Meanwhile, the MCF-7 tumor-bearing mouse treated by the TC/Apt shows the similar fluorescence imaging results to 4T1 tumor tissue (Fig. 6h, i). These data indicate that TC/Apt has the ability to image ATP in vivo.

## Conclusion

In summary, we herein develop a novel kind of fluorescent nanoprobes for ex vivo and in vivo detection and imaging of ATP based on FRET sensing strategy. Specifically, the probes are composed of TC nanosheets linked with ROX-tagged ATP-aptamer through the hydrogen bond and metal chelate interaction between the aptamer and MXenes. Of note, the quenching efficiency of TC sheets against ROX is up to ~ 97%, which is much higher than that of GO nanosheets (~ 90%), leading to a wide dynamic range from 1 μM to 1.5 mM and a low limit of detection (LOD) down to 0.2 μM. Moreover, the as-prepared TC/Apt can be utilized as fluorescent probes for the imaging and sensing of intracellular ATP in living cancer cells treated by Ca^2+^ or etoposide. Furthermore, the TC/Apt probes are capable for detecting the content of ATP in body fluids (e.g., mouse serum, mouse urine, and human serum) and imaging the intracellular ATP in mouse tumor model. Taken together, our findings suggest new opportunities for the treatment of diseases related to abnormal fluctuation of ATP concentration.

## Supplementary Information

**Additional file 1: Table S1.** Comparison of analytical performances of current two-dimensional fluorescent probes. **Figure S1.** TEM images, SEM images, size distribution and DLS of TC and TC/Apt. **Figure S2.** The fluorescence stability of TC/Apt at different storage temperature. **Figure S3.** The pH stability of TC/Apt. **Figure S4.** Hydrogen bond analysis of TC/Apt probes. **Figure S5.** Zeta potential of TC/Apt. **Figure S6.** Thermogravimetric analysis of TC/Apt. **Figure S7.** Agarose gel electrophoresis analysis of free Apt-ROX, TC/Apt without treatment and TC/Apt treated with ATP of different concentrations (*e.g.,* 0.8 and 1.6 mM). **Figure S8.** The fluorescence stability of TC/Apt treated by different bases. **Figure S9.** The fluorescence stability of TC/Apt treated by different ions. **Figure S10.** The fluorescence stability of TC/Apt treated by different amines. **Figure S11.** Cytotoxicity evaluation of the resultant TC/Apt-based probes. **Figure S12.** Energy-dependent endocytosis of TC/Apt. **Figure S13.** Intracellular localization of TC/Apt with different incubation time. **Figure S14.** Intracellular distribution of TC/Apt. **Figure S15.** Confocal images and reconstitution of HeLa and MCF-7 cells treated with TC/Apt. **Figure S16.** Fluorescence cellular imaging of ATP in 4T1 cells with different treatments.
