# Supplementary material for: Ex vivo and in vivo fluorescence detection and imaging of adenosine triphosphate
Source: J Nanobiotechnology. 2021 Jun 22;19:187. doi: 10.1186/s12951-021-00930-4 (PMC8220756; doi:10.1186/s12951-021-00930-4)
Supplement: Supplementary file 1 — Additional file 1: Table S1. Comparison of analytical performances of current two-dimensional fluorescent probes. Figure S1. TEM images, SEM images, size distribution and DLS of TC and TC/Apt. Figure S2. The fluorescence stability of TC/Apt at different storage temperature. Figure S3. The pH stability of TC/Apt. Figure S4. Hydrogen bond analysis of TC/Apt probes. Figure S5. Zeta potential of TC/Apt. Figure S6. Thermogravimetric analysis of TC/Apt. Figure S7. Agarose gel electrophoresis analysis of free Apt-ROX, TC/Apt without treatment and TC/Apt treated with ATP of different concentrations (e.g., 0.8 and 1.6 mM). Figure S8. The fluorescence stability of TC/Apt treated by different bases. Figure S9. The fluorescence stability of TC/Apt treated by different ions. Figure S10. The fluorescence stability of TC/Apt treated by different amines. Figure S11. Cytotoxicity evaluation of the resultant TC/Apt-based probes. Figure S12. Energy-dependent endocytosis of TC/Apt. Figure S13. Intracellular localization of TC/Apt with different incubation time. Figure S14. Intracellular distribution of TC/Apt. Figure S15. Confocal images and reconstitution of HeLa and MCF-7 cells treated with TC/Apt. Figure S16. Fluorescence cellular imaging of ATP in 4T1 cells with different treatments. [file 12951_2021_930_MOESM1_ESM.doc]

**Supplementary Information**

*Ex vivo* and *In vivo* Fluorescence Detection and Imaging of Adenosine Triphosphate

Binbin Chu, ^‡, 2^ Ajun Wang, ^‡, 1^ Liang Cheng, ^2^ Runzhi Chen, ^2^ Huayi Shi, ^2^ Bin Song, ^2^ Fenglin Dong ^*, 1^ Houyu Wang, ^*, 2^ and Yao He ^*, 2^

*^1^ The First Affiliated Hospital of Soochow University, Soochow University, Suzhou, Jiangsu 215006, China*

*^2^ Laboratory of Nanoscale Biochemical Analysis, Jiangsu Key Laboratory for Carbon-Based Functional Materials and Devices, Institute of Functional Nano & Soft Materials (FUNSOM), Soochow University, Suzhou, Jiangsu 215123, China*

*E-mail: fldong@suda.edu.cn (Fenglin Dong); houyuwang@suda.edu.cn (Houyu Wang); yaohe@suda.edu.cn (Yao He)*

*‡ These authors contributed equally.*

**List of Contents**

**Table S1.** Comparison of analytical performances of current two-dimensional fluorescent probes.

**Figure S1.** TEM and SEM images and DLS of TC and TC/Apt.

**Figure S2.** The fluorescence stability of TC/Apt at different storage temperature.

**Figure S3.** The pH stability of TC/Apt.

**Figure S4.** Hydrogen bond analysis of TC/Apt.

**Figure S5.** Zeta potential of TC/Apt.

**Figure S6.** Thermogravimetric analysis of TC/Apt.

**Figure S7.** Agarose gel electrophoresis analysis.

**Figure S8.** The fluorescence stability of TC/Apt treated by different bases.

**Figure S9.** The fluorescence stability of TC/Apt treated by different ions.

**Figure S10.** The fluorescence stability of TC/Apt treated by different amines.

**Figure S11.** Cytotoxicity evaluation of the resultant TC/Apt-based probes.

**Figure S12.** Energy-dependent endocytosis of TC/Apt.

**Figure S13.** Intracellular localization of TC/Apt with different incubation time.

**Figure S14.** Intracellular distribution of TC/Apt.

**Figure S15.** Confocal images and reconstitution of HeLa and MCF-7 cells treated with TC/Apt.

**Figure S16.** Fluorescence cellular imaging of ATP in 4T1 cells with different treatments.

**1. Reagents and apparatus**

The Ti_3_AlC_2_ starting material, HF (40%, purity >98%), and TMAOH were purchased from Beijing Forsman Scientific, Sigma-Aldrich, and Baimingwei Company, respectively. DNA oligonucleotides (DNA sequences: ACCTGGGGGAGTATTGCGGAGGAAGGT-ROX) were synthesized and purified by Sangon Biotechnology (Shanghai, China). Adenosine Triphosphate (ATP), cytidine triphosphate (CTP), guanosine triphosphate (GTP), and uridine Triphosphate (UTP) were bought from Sangon Biotechnology (Shanghai, China). Adenosine monophosphate (AMP) was purchased from Zhongchu Biological Technology Co., Ltd (Suzhou, China). Many kinds of amino acids (e.g., Asn, Asp, Cys, Gln, Glu, His, Met, Phe, Pro, Ser, Thr, Trp, Gly, Arg, and Lys) and chemicals (e.g., Ca^2+^, K^+^, Cl^-^, Mg^2+^, Na^+^, ClO^-^, Al^3+^, Cd^2+^, Cu^2+^, Br^−^, Fe^2+^, Mn^2+^, Hg^2+^, Ni^+^, Zn^2+^, I^−^, ClO_4_^−^, Fe^3+^, Pb^2+^, D-glucose, dopamine, H_2_O_2_, and vitamin C) were obtained from Sinopharm Chemical Reagent Co., Ltd (Shanghai, China). Dulbecco’s modified eagle media (DMEM), Roswell Park Memorial Institute 1640 (RPMI-1640) media, fetal bovine serum (FBS), penicillin/streptomycin (100 μg/mL), and phosphate buffered saline (PBS) solution were obtained from Invitrogen Corporation (Life Technologies, Shanghai, China). Etoposide was bought from Sigma-Aldrich (Shanghai, China). Graphene oxide (GO) was bought from the J&K Scientific Co., Ltd. All chemicals were analytical grade and used without additional purification. All solutions were prepared using distilled water (Millipore).

The morphologies of TC and TC/Apt were characterized through transmission electronic microscopy (TEM) and high-resolution TEM (HRTEM), using an electron microscope (Philips CM 200) with 200 kV. SEM images were collected by a scanning electron microscopy (SEM) (FEI Quanta 200F) equipped with energy-dispersive X-ray (EDX) spectroscopy. A Raman microscope (HR800, Horiba Jobin Yvon, France) equipped with a 633 nm He-Ne laser (20 mW, polarized 500:1) was employed for analyze of Raman spectra. A 750 UV-vis near-infrared spectrophotometer (Perkin-Elmer lambda) was used for measurement of UV-vis absorption spectra. A spectro-fluorimeter (HORIBA JOBIN YVON FLUORMAX-4) was employed for recording photoluminescence (PL). Delsa™ nano submicron particle size and Zeta potential particle analyzer (Beckman Coulter, Inc) was employed for the analysis of dynamic light scattering (DLS) and zeta potentials. A Delta 320 pH-meter (Mettler-Toledo Instruments (Shanghai) Co., China) was used for pH measurements. Fluorescence imaging experiments were performed on a confocal laser scanning microscope (Leica, TCS-SP5 II). In vivo fluorescence image of these mice with different treatment is captured by a Maestro EX in vivo fluorescence imaging system (CRi, Inc.).

**2. Comparison of analytical performances/characterizations of current two-dimensional (2D) nanomaterials-based fluorescent ATP probes**

**Table S1.** Comparison of analytical performances of current 2D nanomaterials-based fluorescent probes for detection of ATP.

| Method | QE ^c^ | Detection limit | Detection range | Cell imaging ^d^ | *In vivo* imaging | Real samples ^e^ | Refs |
| --- | --- | --- | --- | --- | --- | --- | --- |
| Fluorescence based on GO ^a^ | Not given | Not given | 0.01-2.5 mM | Yes | Not given | Not given | 1 |
| Fluorescence based on GO | Not given | Not given | Not given | Yes | Not given | Not given | 2 |
| Fluorescence based on GO | Not given | 0.028 mM | 0.125-2 mM | Yes | Not given | Not given | 3 |
| Fluorescence based on GO | Not given | 0.5 μM | 0.01-3 mM | Yes | Not given | Not given | 4 |
| Fluorescence based on GO | Not given | 5 nM | 0.01-1 μM | Not given | Not given | Not given | 5 |
| Fluorescence based on GO | Not given | 31 nM | 50-500 nM | Not given | Not given | Not given | 6 |
| Fluorescence based on GO | Not given | 0.42 nM | 1-200 nM | Not given | Not given | Yes | 7 |
| Fluorescence based on MoS_2_ | Not given | 4 μM | 0.01-2 mM | No | Not given | Not given | 8 |
| Fluorescence based on MoS_2_ | Not given | Not given | 0.005-3 mM | Yes | Not given | Not given | 9 |
| Fluorescence based on MoS_2_ | Not given | 34.4 nM | 0.07-26.7 μM | Not given | Not given | Yes | 10 |
| **Fluorescence based on GO** | **90% ^9^** | **0.46 mM** | **0.5-1.5 mM** | **No** | **No** | **No** | **This work** |
| **Fluorescence based on TC** ^b^ | **97%** | **0.2 μM** | **0.001-1.5 mM** | **Yes** | **Yes** | **Yes** | **This work** |

^a^ GO: graphene oxide; ^b^ TC: titanium carbide nanosheets; ^c^ QE: quenching efficiency; ^d^ Cell imaging: *in vitro* imaging of intracellular ATP in live cells. ^e^ Real samples includes bodily fluid (e.g., mouse serum, rat urine, and human serum) and so on.

The comparison of analytical performance of several 2D nanomaterials-based fluorescent probes for ATP assay is summarized in the **Table S1**. Brief discussions are as follow,

1. As revealed in these recently reported works, the resultant TC nanosheets feature stronger fluorescence quenching capacity than the conventional GO to some extent. ^9^ In order to experimentally compare the quenching efficiency (QE) of TC nanosheets against Apt-ROX in our current systems, the commercial GO is selected for a comparison in our experiment. Typically, the corresponding QE of commercial GO is calculated as ~90%, which is similar to QE value in previous work. ^9^ On the contrary, TC nanosheets reach higher fluorescence QE value of ~97%, favoring in superior detection range and detection limit.
2. In comparison to mostly reported GO- or MoS_2_-based ATP probes, ^1-4, 8, 9^ the TC/Apt in this work possess superior detection limit (i.e., 0.2 μM).
3. The TC/Apt probes simultaneously feature considerable detection range for *in vitro* and *in vivo* imaging analysis of the intracellular ATP. ^1-4, 9, 11-14^ While our TC/Apt probes have relatively lower detection limit compared to a few GO-based fluorescent probes mentioned in previous works, ^5, 6^ the TC/Apt probes in this work can be further utilized for *in vitro* and *in vivo* imaging and semiquantitative detection of intracellular ATP, which are not provided in corresponding previous works.
4. The prepared TC/Apt probes are feasible for accurate discrimination of ATP with a varied concentration spiked in the bodily fluid (e.g., mouse serum, rat urine, and human serum ^10^).

To be summarized in **Table S1**, the presented 2D TC/Apt-based fluorescent ATP probes simultaneously possess superior detection limit, considerable detection range, and feasibility for *in vitro* and *in vivo* imaging and semiquantitative detection of intracellular ATP. More importantly, the prepared TC/Apt probes is feasible for the accurate discrimination of ATP with a varied concentrations spiked in the bodily fluid (e.g., mouse serum, mouse urine, and human serum).

**3. Characterization of TC nanosheets and TC/Apt**

**3.1 TEM images, SEM images, size distribution and DLS of TC and TC/Apt**

Samples for TEM analysis were prepared by placing 10 μL of the TC nanosheets or TC/Apt solution on the carbon-coated copper grid and then dryed at room temperature. ^15, 16^ Meanwhile, these samples for SEM analysis were prepared by placing 20 μL of the pure TC nanosheets or TC/Apt solution on the silicon chip and then dryed at room temperature.


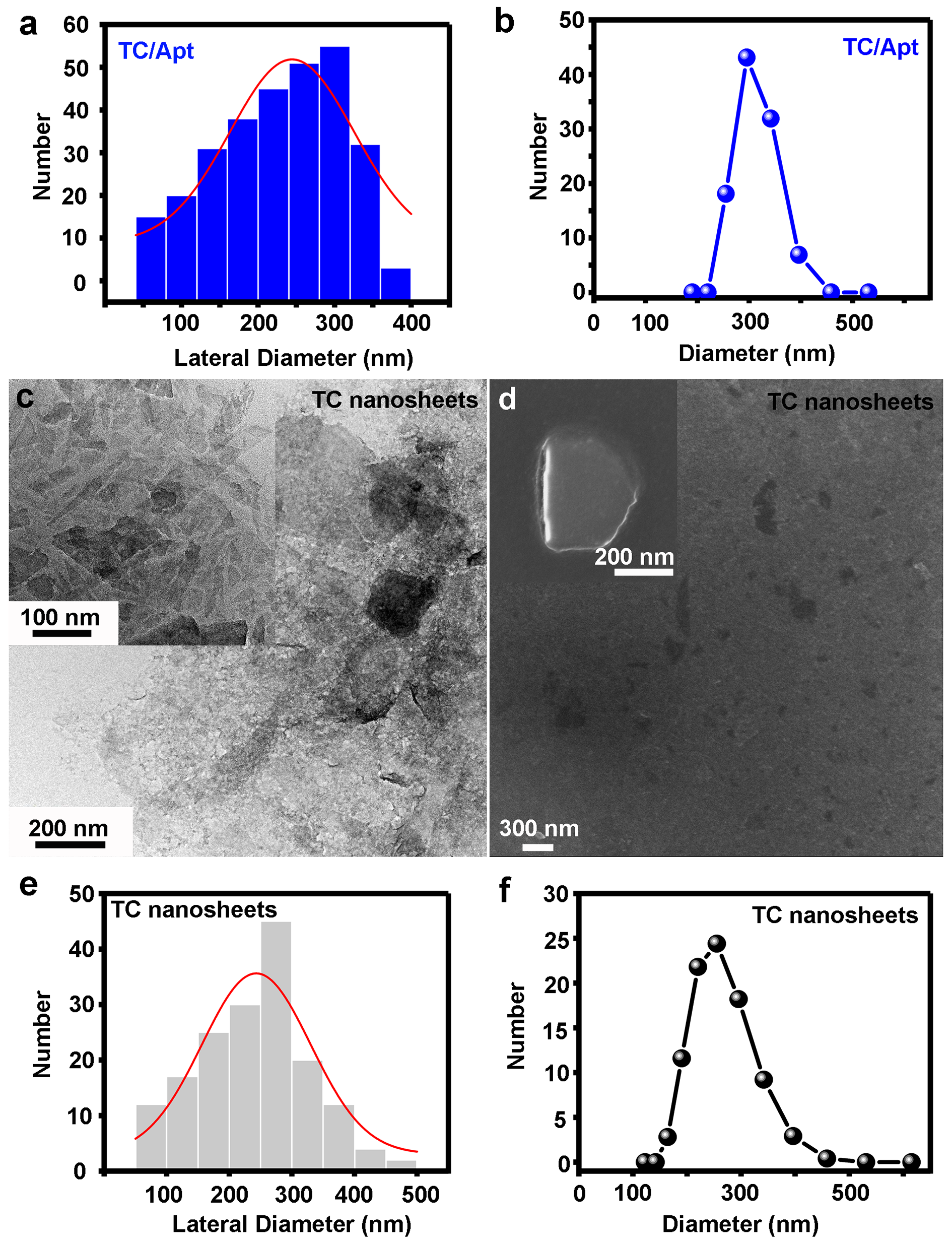


**Figure S1*.* TEM images, SEM images, size distribution and DLS analysis of TC nanosheets and** **TC/Apt nanoprobes.** **a** Size distribution of TC/Apt nanoprobes. **b** DLS data of TC/Apt nanoprobes. **c** TEM and HRTEM images of pure TC nanosheets. **d** The SEM images of pure TC nanosheets. **e** Size distribution of TC nanosheets. **f** DLS data of TC nanosheets.

As shown in **Figure S1**, TC nanosheets present sheet-like structure with ~200-300 nm lateral dimensions with the good dispersibility.

**3.2 Fluorescence stability of TC/Apt**


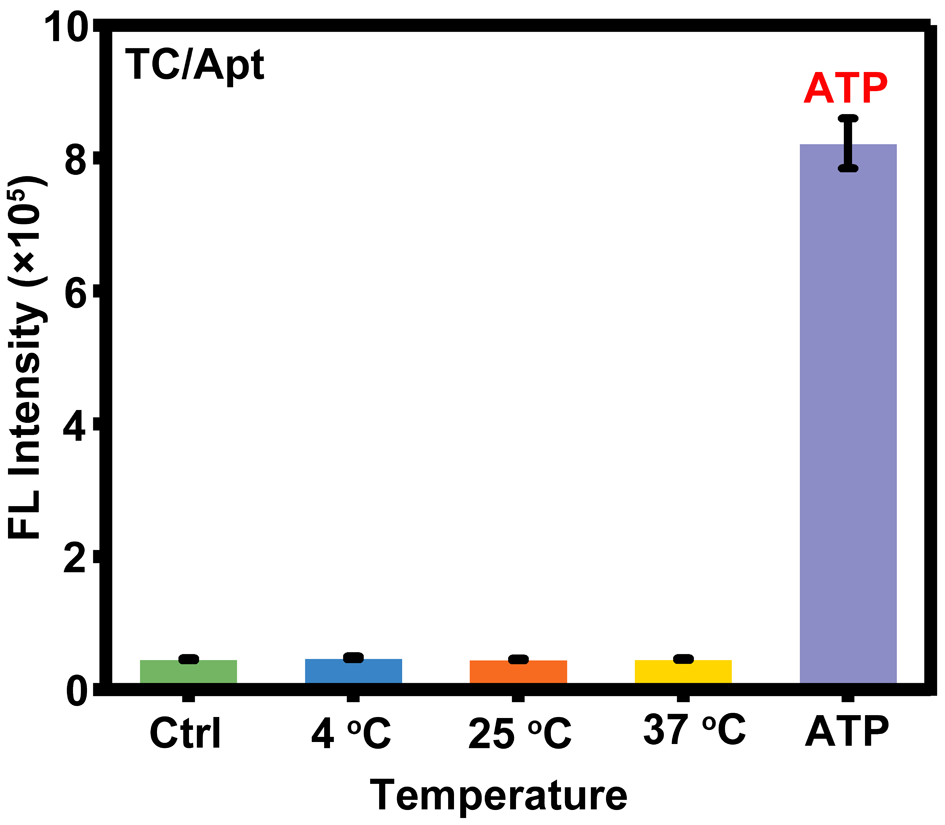


**Figure S2*.* The fluorescence stability of the TC/Apt at different storage temperature.** Corresponding histograms of the fluorescence intensity of the resultant TC/Apt at 610 nm versus storage temperature (e.g., 4, 25, and 37 ^o^C). The untreated TC/Apt solutions are set as control groups. All error bars show the standard deviation determined from three independent assays.

As shown in the **Figure S2**, the as-prepared TC/Apt possess excellent fluorescence stability at different storage temperature with no specific protection, revealing that specificity of the TC/Apt is not influenced by different temperatures (e.g., 4, 25, and 37 ^o^C).


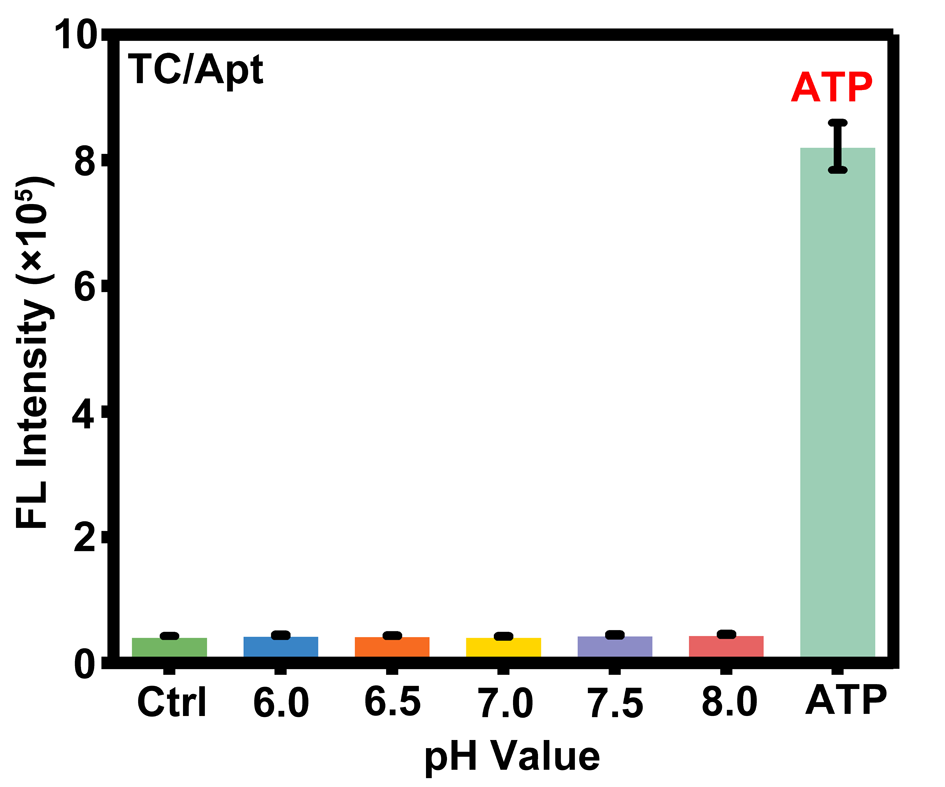


**Figure S3*.* pH stability of TC/Apt.** Corresponding histograms of the fluorescence intensity of the resultant TC/Apt at 610 nm versus various pH values (e.g., 6.0, 6.5, 7.0, 7.5, and 8.0). The untreated TC/Apt solutions are set as control groups. All error bars show the standard deviation determined from three independent assays.

As shown in the **Figure S3**, the as-prepared TC/Apt possess excellent fluorescence stability at different pH values (e.g., 6.0, 6.5, 7.0, 7.5, and 8.0), revealing that specificity of the TC/Apt is not influenced by different pH values of solutions (e.g., 6.0, 6.5, 7.0, 7.5, and 8.0).

**3.3 Hydrogen bond analysis of TC/Apt probes**


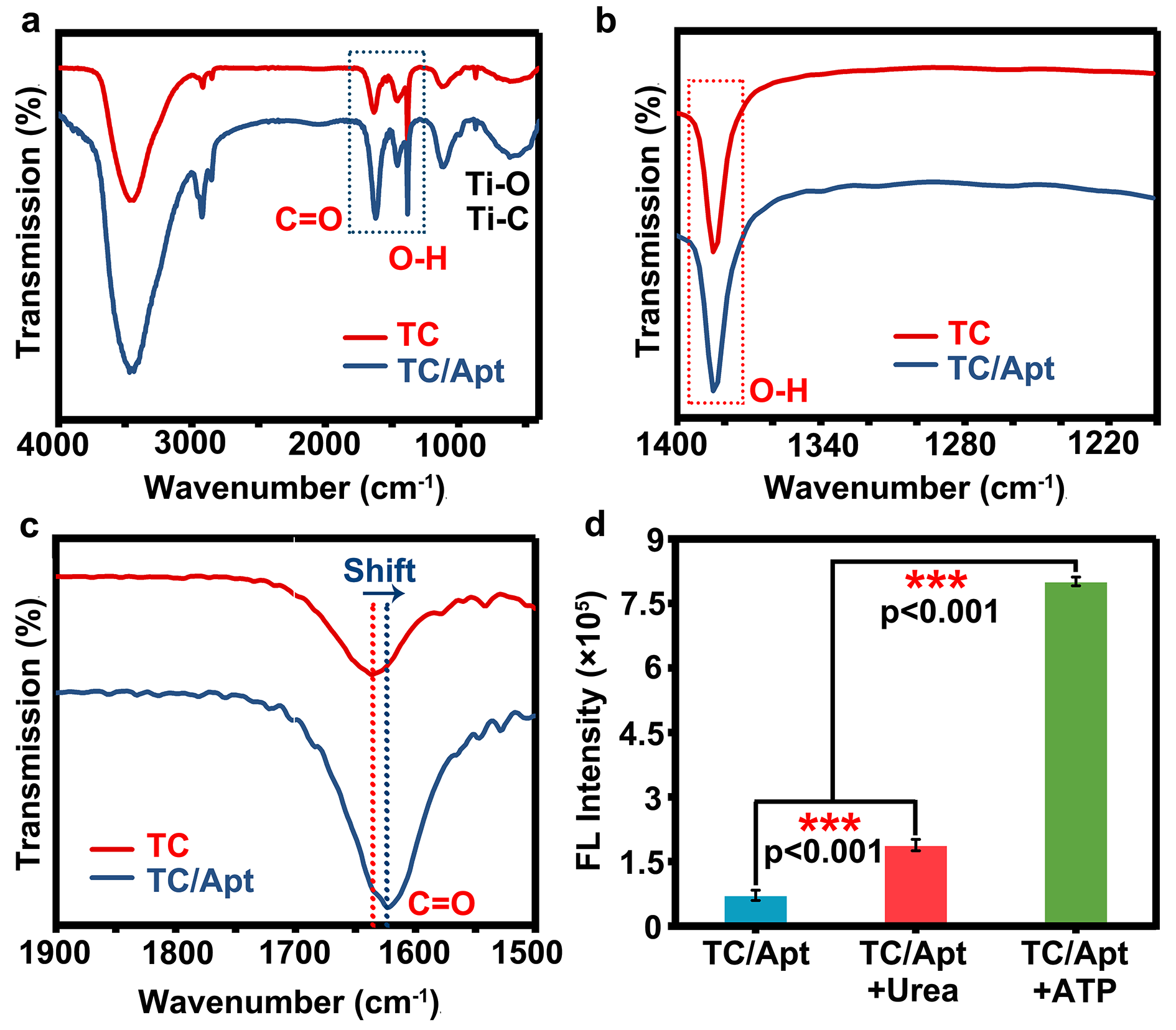


**Figure S4*.* Hydrogen bond analysis of the resultant TC/Apt.** (**a-c**) The FTIR spectra of TC nanosheets (red line) and TC/Apt (blue line). (**d**) Fluorescence intensities of the TC/Apt probes in different environments (pure water, urea, and ATP). *** represents p < 0.001.

The FTIR spectroscopy of TC nanosheets and TC/Apt are measured in **Figures S4a-c**, respectively. In particular, several identical absorption peaks in the range of ~400-500 cm^−1^ observed in both TC nanosheets and TC/Apt samples are assigned to Ti-C and Ti-O (**Figure S4a**). As shown in **Figure S4b**, the peak of aptamer including O-H at ~1383 cm^-1^, which is covered by Mxenes. As shown in **Figure S4c**, compared with the pure TC nanosheets, the TC/Apt probes show a shoulder peak of the carbonyl (C=O) stretching band at ~1640 cm^-1^ with a slight peak shift to short wavenumber. ^17^ In addition, a high concentration of urea can destroy the hydrogen bond, which is chosen for confirming the existence of a hydrogen bond. ^18^ As further exhibited in **Figure S4d**, the fluorescence recovery slightly increases after adding urea (p < 0.001), indicating the existence of a hydrogen bond between TC nanosheets and Apt-ROX. Taken together, these results demonstrate that The Apt-ROX can be facilely and selectively adsorbed onto TC nanosheets by hydrogen bond between aptamer and TC. ^17, 18^

**3.4 Zeta potential of TC/Apt**


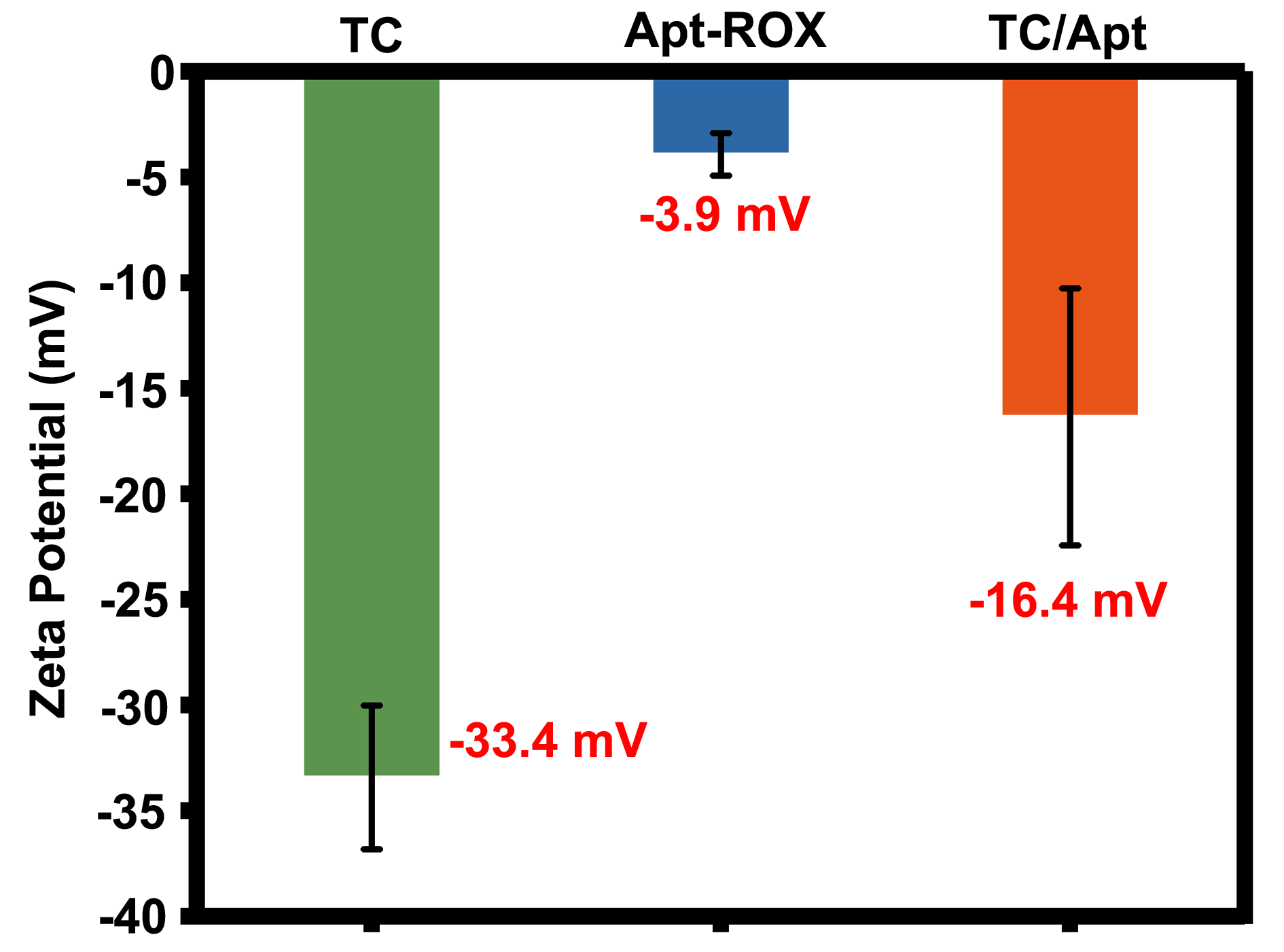


**Figure S5*.* Zeta potential of TC/Apt.** Zeta potentials of TC nanosheets, Aptamer-ROX, and TC/Apt.

Zeta potentials of deionized water, Aptamer-ROX, TC nanosheets, and TC/Apt are presented in **Figure S5**. When Aptamer-ROX is linked to the surface of TC nanosheets, the zeta potential decreases from higher negative charge (ca. -33.4 mV) to lower negative charge (ca. -16.4 mV).

**3.5 Thermogravimetric analysis of TC/Apt**


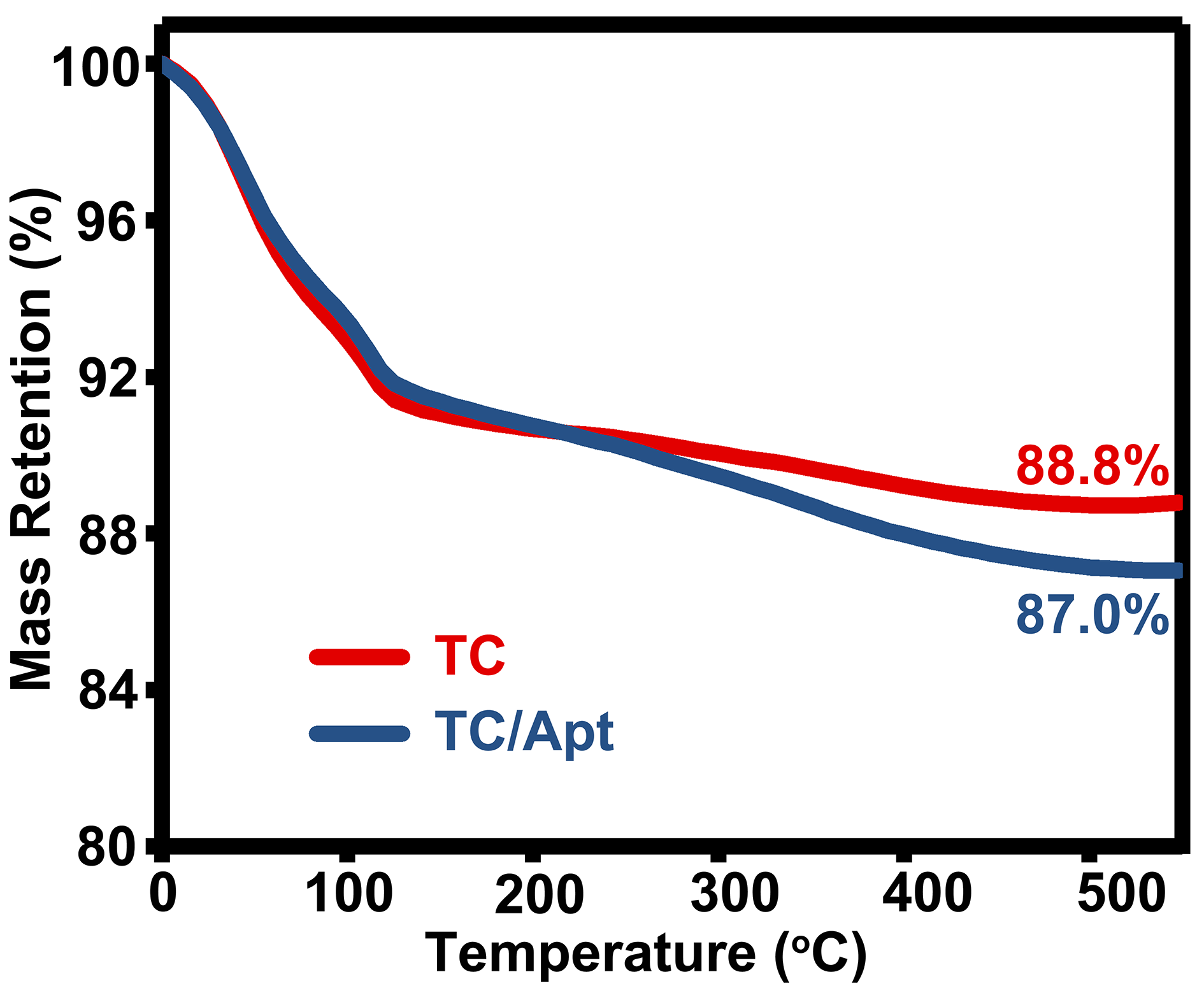


**Figure S6*.* Thermogravimetric analysis of TC/Apt.** The thermogravimetric analysis of TC nanosheets (red line) and TC/Apt (blue line).

As shown in **Figure S6**, the weight loss percentage of TC/Apt and TC nanosheets is calculated as ~13% and ~11.2%, respectively, at the temperature of ~550 ^o^C. The smaller weight loss percentage of TC nanosheets is resulted from the burning of carbonous species; comparatively, the TC/Apt probes feature relatively larger weight loss percentage, which is ascribed to both burning of carbonous species in TC nanosheets and carbonous residues contained in Apt-ROX. These results provide additional evidence that Apt-ROX is incorporated with TC nanosheets to from TC/Apt probes and the mass ratio of Apt-ROX in TC/Apt can be calculated as ~1.8%.

**3.6 Agarose gel electrophoresis analysis**


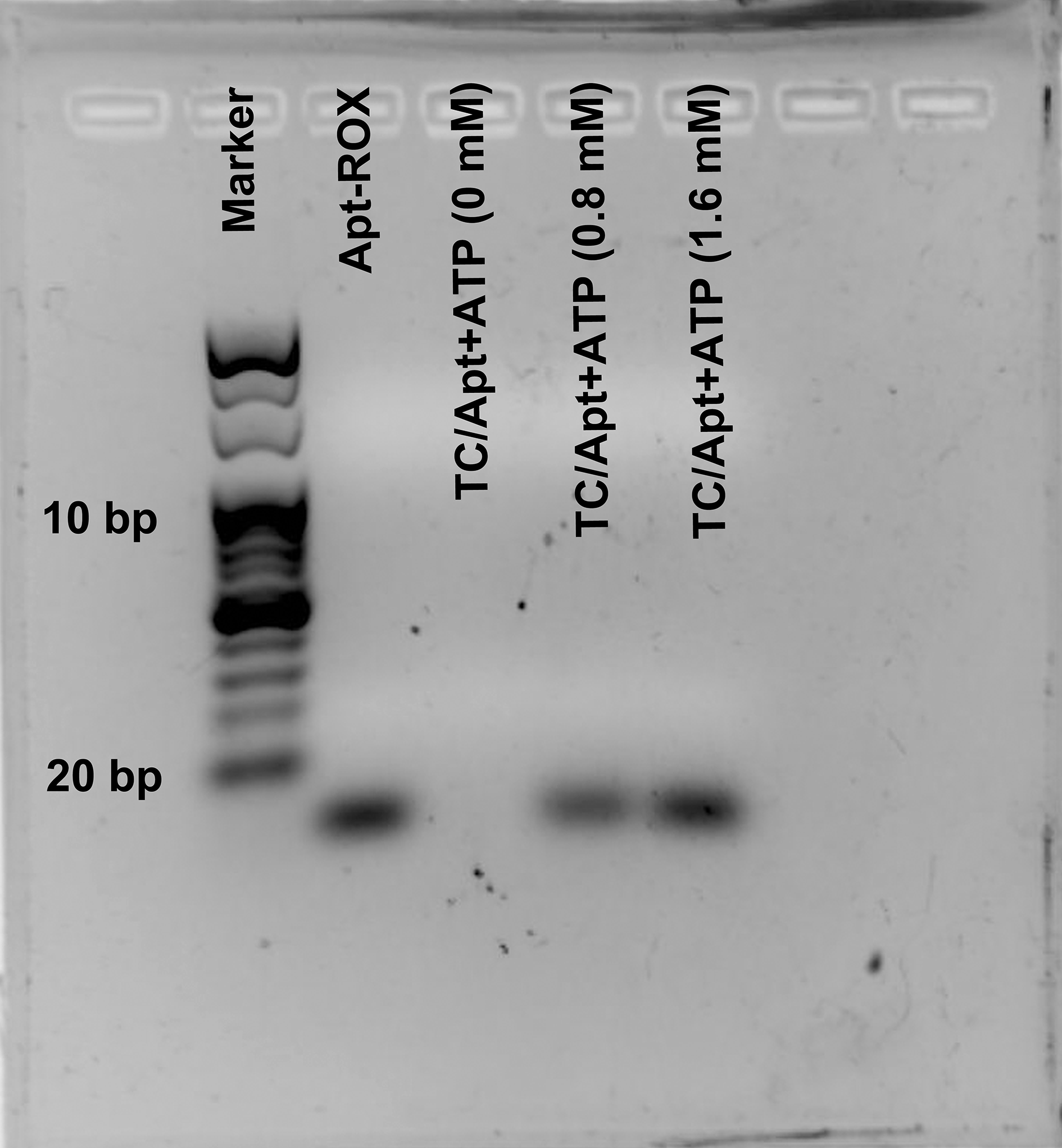


**Figure S7*.*** **Agarose gel electrophoresis analysis of free aptamer in supernatant of TC/Apt solutions after the treatment of ATP.** The agarose gel electrophoresis analysis of free Apt-ROX, TC/Apt without treatment and TC/Apt treated with ATP of different concentrations (e.g., 0.0, 0.8 and 1.6 mM).

As shown in **Figure S7**, no obvious free aptamer bands in supernatant of TC/Apt solutions without treatment of ATP (0 mM); more and more free aptamer bands can be detected in the supernatant of TC/Apt solutions after the different treatments of ATP (0.8 and 1.6 mM), which is similar to that of free Apt-ROX. Thus, these results demonstrate that the free ATP are able to desorb Apt-ROX from TC/Apt probes by specifically targeting the Apt-ROX and changing conformations of Apt-ROX.

**4. Interference Study**

To evaluate the specificity of the resultant TC/Apt, the prepared solutions containing 200 μg/mL TC/Apt mixed with 1 mM uridine triphosphate (UTP), 1 mM cytidine triphosphate (CTP), 1 mM guanosine triphosphate (GTP), or 1 mM adenosine monophosphate (AMP) was tested by the measurement of fluorescence emission intensities under 545-nm excitation. In addition, the effects of different interference ions (e.g., 150 mM K^+^, 150 mM Cl^-^, 2 mM Mg^2+^, 2 mM Ca^2+^, 10 mM Na^+^, 0.1 mM ClO^-^, 0.1 mM Al^3+^, 0.1 mM Cd^2+^, 0.1 mM Cu^2+^, 0.1 mM Br^−^, 0.1 mM Fe^2+^, 0.1 mM Mn^2+^, 0.1 mM Hg^2+^, 0.1 mM Ni^+^, 0.1 mM Zn^2+^, 0.1 mM I^−^, 0.1 mM ClO_4_^−^, 0.1 mM Fe^3+^ and 0.1 mM Pb^2+^) on the fluorescence intensity of the as-prepared TC/Apt (100 μg/mL) were investigated in PBS buffers. In addition, the effects of various species (e.g., 0.5 mM Asn, 0.5 mM Asp, 1 mM Cys, 1 mM Gln, 0.5 mM Glu, 1 mM His, 0.5 mM Met, 0.5 mM Phe, 1 mM Pro, 1 mM Ser, 0.5 mM Thr, 0.5 mM Trp, 1 mM Gly, 1 mM Arg, 1 mM Lys, 5 mM D-glucose, 0.1 mM dopamine, 0.1 mM H_2_O_2_, 1 mM bovine serum albumin (BSA), and 0.5 mM vitamin C) on the fluorescence intensity of the as-prepared TC/Apt (100 μg/mL) were investigated in PBS buffers. The fluorescence intensity at 610 nm of the TC/Apt was recorded.


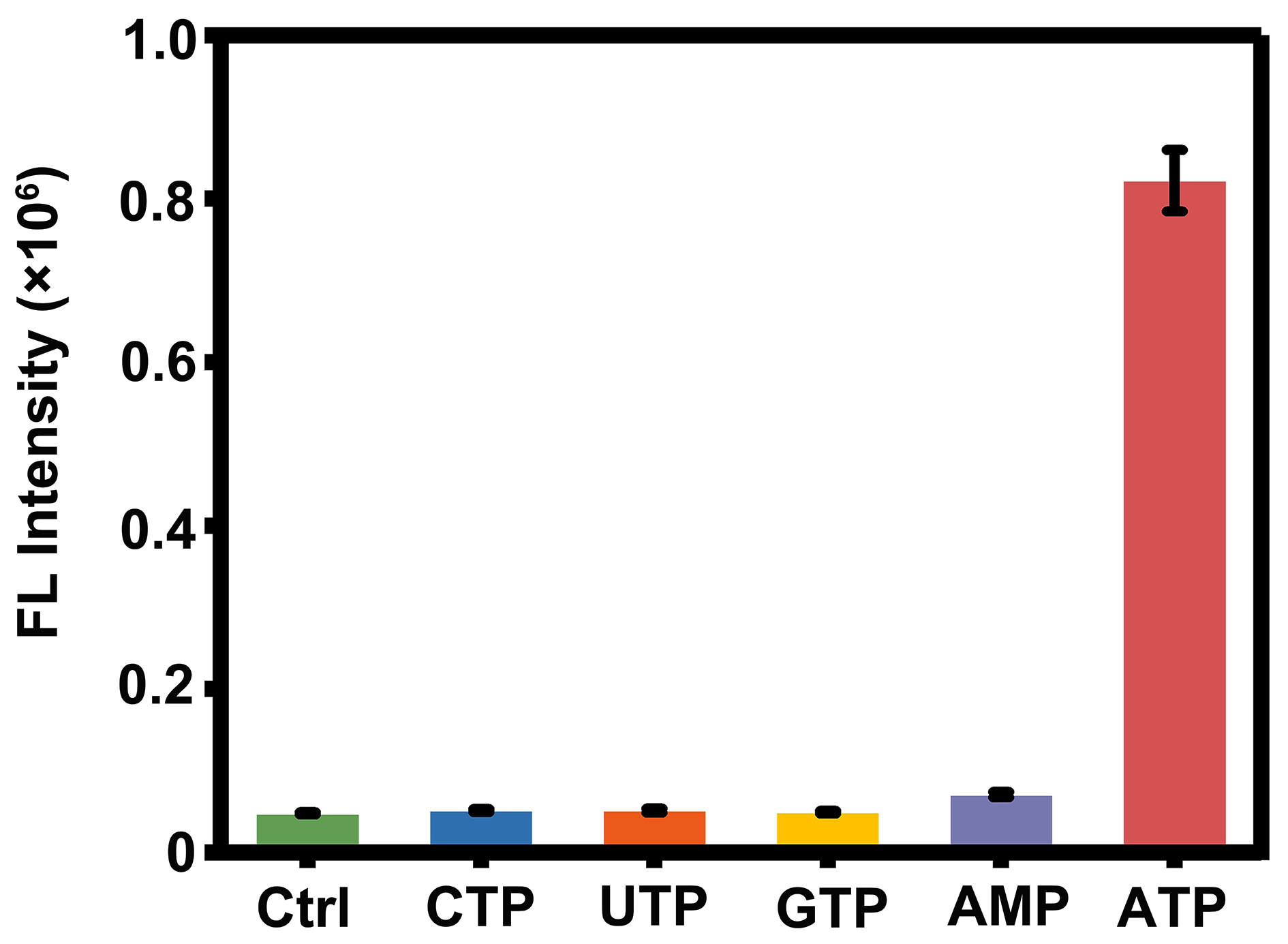


**Figure S8*.*** **The fluorescence stability of TC/Apt treated by different bases.** Histograms of fluorescence intensity of 200 μg/mL TC/Apt treated with1 mM CTP, 1 mM UTP, 1 mM GTP, 1 mM AMP or 1 mM ATP. The measurements are repeated for three times.


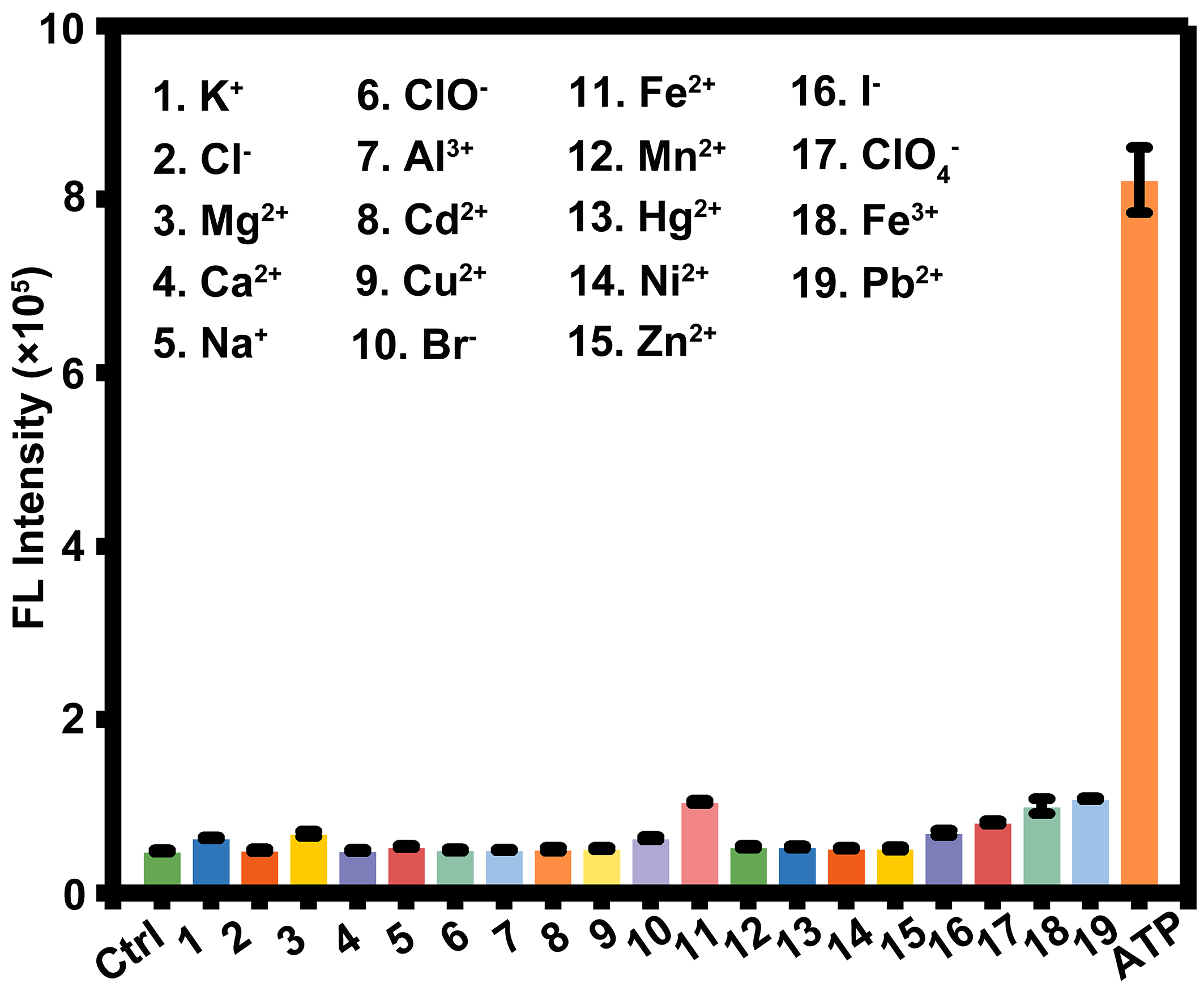


**Figure S9*.*** **The fluorescence stability of TC/Apt treated by different ions.** The fluorescence intensity at 610 nm in the absence (control) and in the presence of different ions (e.g., 150 mM K^+^, 150 mM Cl^-^, 2 mM Mg^2+^, 2 mM Ca^2+^, 10 mM Na^+^, 0.1 mM ClO^-^, 0.1 mM Al^3+^, 0.1 mM Cd^2+^, 0.1 mM Cu^2+^, 0.1 mM Br^−^, 0.1 mM Fe^2+^, 0.1 mM Mn^2+^, 0.1 mM Hg^2+^, 0.1 mM Ni^+^, 0.1 mM Zn^2+^, 0.1 mM I^−^, 0.1 mM ClO_4_^−^, 0.1 mM Fe^3+^ and 0.1 mM Pb^2+^). The effects of different ions are examined in pH 7.4 PBS buffer. The concentration of the TC/Apt is 100 μg/mL. The measurements are repeated for three times.


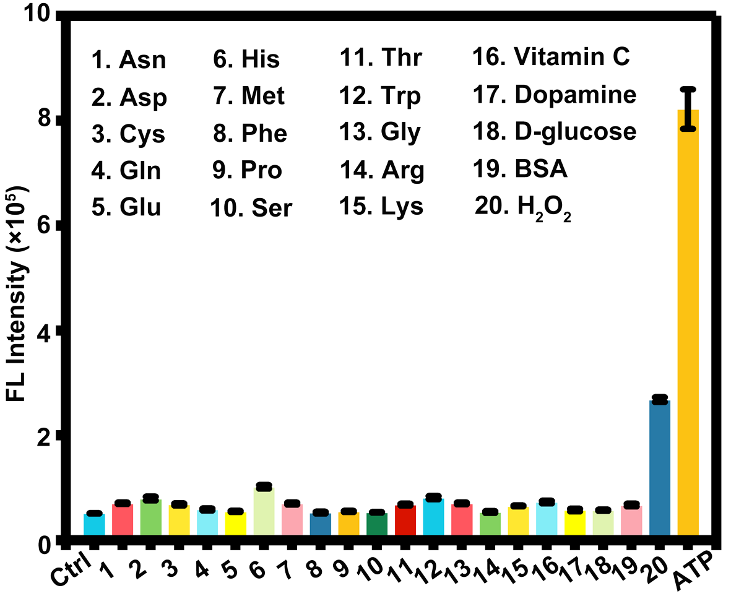


**Figure S10*.* The fluorescence stability of TC/Apt treated by different amines.** The fluorescence intensity at 610 nm in the absence (control) and in the presence of various amine acids (e.g., 0.5 mM Asn, 0.5 mM Asp, 1 mM Cys, 1 mM Gln, 0.5 mM Glu, 1 mM His, 0.5 mM Met, 0.5 mM Phe, 1 mM Pro, 1 mM Ser, 0.5 mM Thr, 0.5 mM Trp, 1 mM Gly, 1 mM Arg, 1 mM Lys, 5 mM D-glucose, 0.1 mM dopamine, 0.1 mM H_2_O_2_, 1 mM bovine serum albumin (BSA), and 0.5 mM vitamin C). The concentration of the TC/Apt is 100 μg/mL. All error bars represent the standard deviation obtained from three independent measurements.

As depicted in **Figures S8-S10**, there is no significant statistic variation among interference groups and control group (p> 0.05), suggesting negligible effect of interference bases, cations, anions, and various amine acids on the resultant TC/Apt.

**5. Cytotoxicity assay**

The cytotoxicity of the prepared TC/Apt was evaluated by the standard MTT (3-(4, 5-dimethylthiazolyl-2)-2, 5-diphenyltetrazolium bromide) assay. ^19^ Briefly, three cell lines, i.e., HeLa, MCF-7, and 4T1, were respectively dispersed in 96-well cell-culture plate at a density of 2.0×10^4^ cells/well, and incubated with the TC/Apt at varied concentrations (12.5, 25, 50, 100, and 200 μg/mL) at 37 ^o^C for 12 or 24 h. Then 20 µL stock MTT solution (5 mg/mL) was added to each well, cultured with cells at 37 ^o^C for ~6 h. Afterwards, the cells were lysed by 10% acidified sodium dodecyl sulfate (SDS) or Dimethyl sulfoxide (DMSO). The cell viability was determined through measuring the absorbance of 570 nm via the microplate reader (Bio-Rad 680, U.S.A.). Three independent assays were performed in triplicate for all measurements.


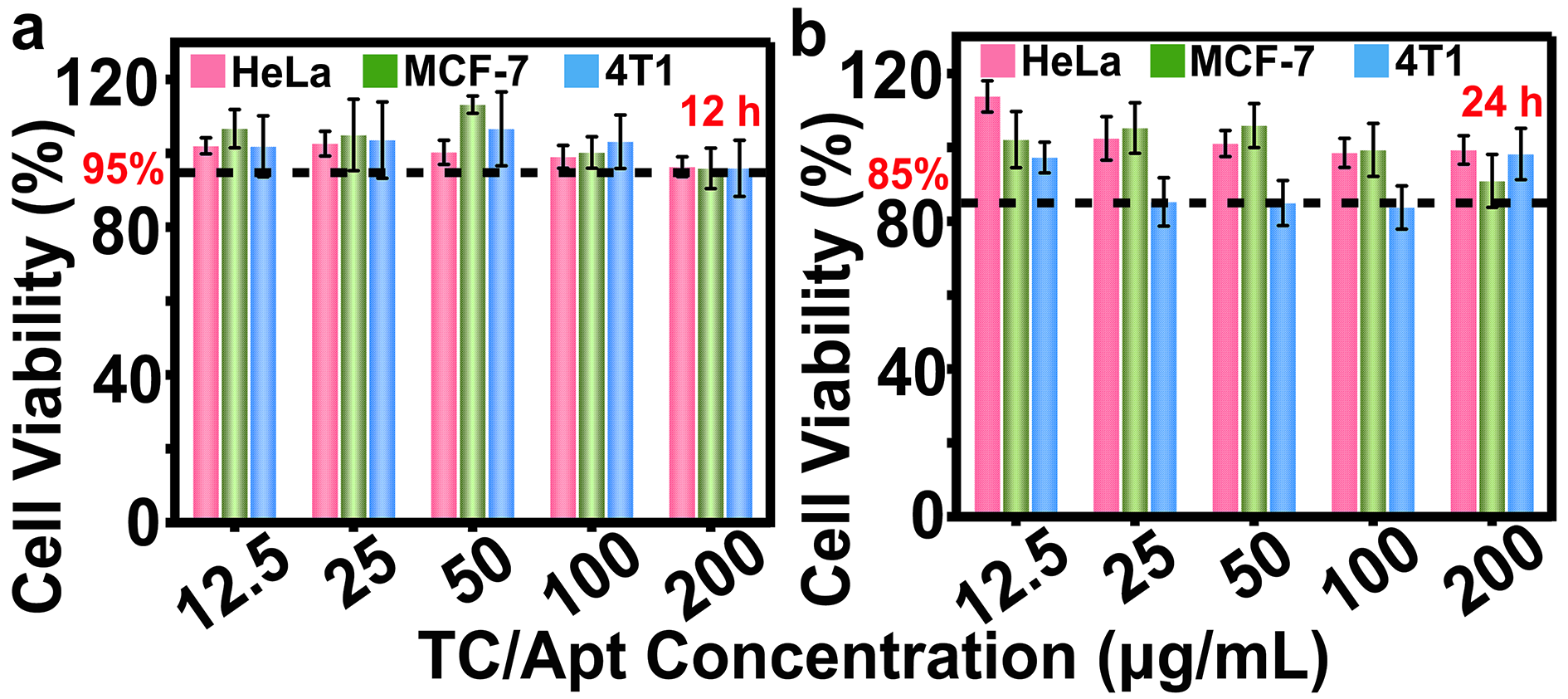


**Figure S11.** **Cytotoxicity evaluation of the resultant TC/Apt-based platform.** These relative MTT assay results of HeLa, MCF-7, and 4T1 cells treated with TC/Apt with different concentrations (e.g., 12.5, 25, 50, 100, and 200 μg/mL) for both 12 h (**a**) and 24 h (**b**). Error bars show standard deviation determined from three independent measurements.

The viabilities of treated cells are above ~85% (**Figure S11**), suggesting low cytotoxicity of the resultant TC/Apt probes.

**6. Energy-dependent endocytosis of TC/Apt**

The HeLa or MCF-7 cells after a 12-h culturing were respectively incubated with 100 μg/mL TC/Apt at 37 ^o^C or 4 ^o^C for another 6 h, respectively. Before monitoring, the resultant cells were washed with PBS (pH 7.4) for three times to remove nonspecifically adsorbed TC/Apt. Fluorescence imaging of these treated cells were performed by a confocal laser scanning microscope (CLSM, Leica, TCS-SP5 II) with 30% power of diode laser. Emission window of TC/Apt was set as 560-650 nm with excitation at 543 nm.


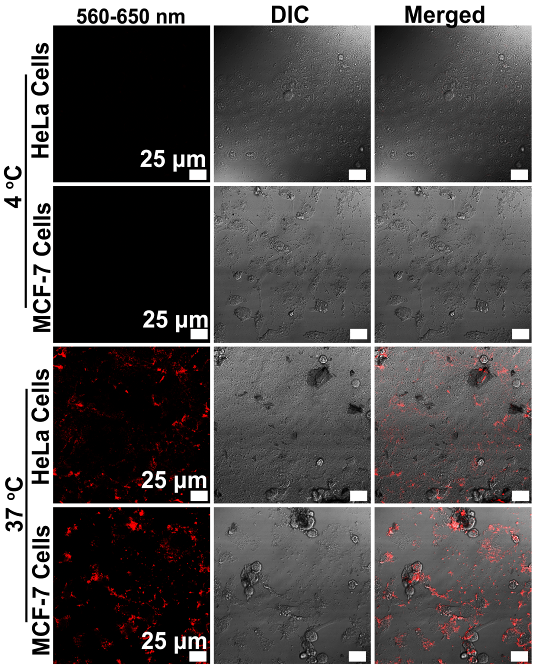


**Figure S12.** **Energy-dependent endocytosis of TC/Apt.** Confocal pictures of HeLa cells and MCF-7 cells treated by TC/Apt for 6 h at 37 ^o^C and 4 ^o^C. Scale bars, 25 μm.

As suggested in **Figure S12**, when the incubation temperature is 37 ^o^C, there are obvious red fluorescence in cytoplasm and no distinct red fluorescence in nucleus. However, there are undetectable red fluorescence in both nucleus and cytoplasm at 4 ^o^C. These mean the TC/Apt enter into cells *via* energy-dependent endocytosis.

**7. Intracellular localization of TC/Apt**

The HeLa or MCF-7 cells after 12-h culturing were respectively incubated with 100 μg/mL TC/Apt at 37 ^o^C for 0, 3, 6, 12, and 24 h. Before monitoring, the resultant cells were washed with PBS (pH 7.4) for three times to remove nonspecifically adsorbed TC/Apt. Fluorescence imaging of these treated cells was performed by CLSM (Leica, TCS-SP5 II) with 30% power of diode laser. Emission window of TC/Apt was set as 560-650 nm with excitation at 543 nm. Region of interest (ROI) was employed for the quantitative assessments of fluorescence intensity of TC/Apt, which was calculated by the commercial image analysis software (Leica Application Suite Advanced Fluorescence Lite (short for LAS AF Lite)) and the software of ImageJ (NIH Image; http;//rsbweb.nih.gov/ij/). ^20^


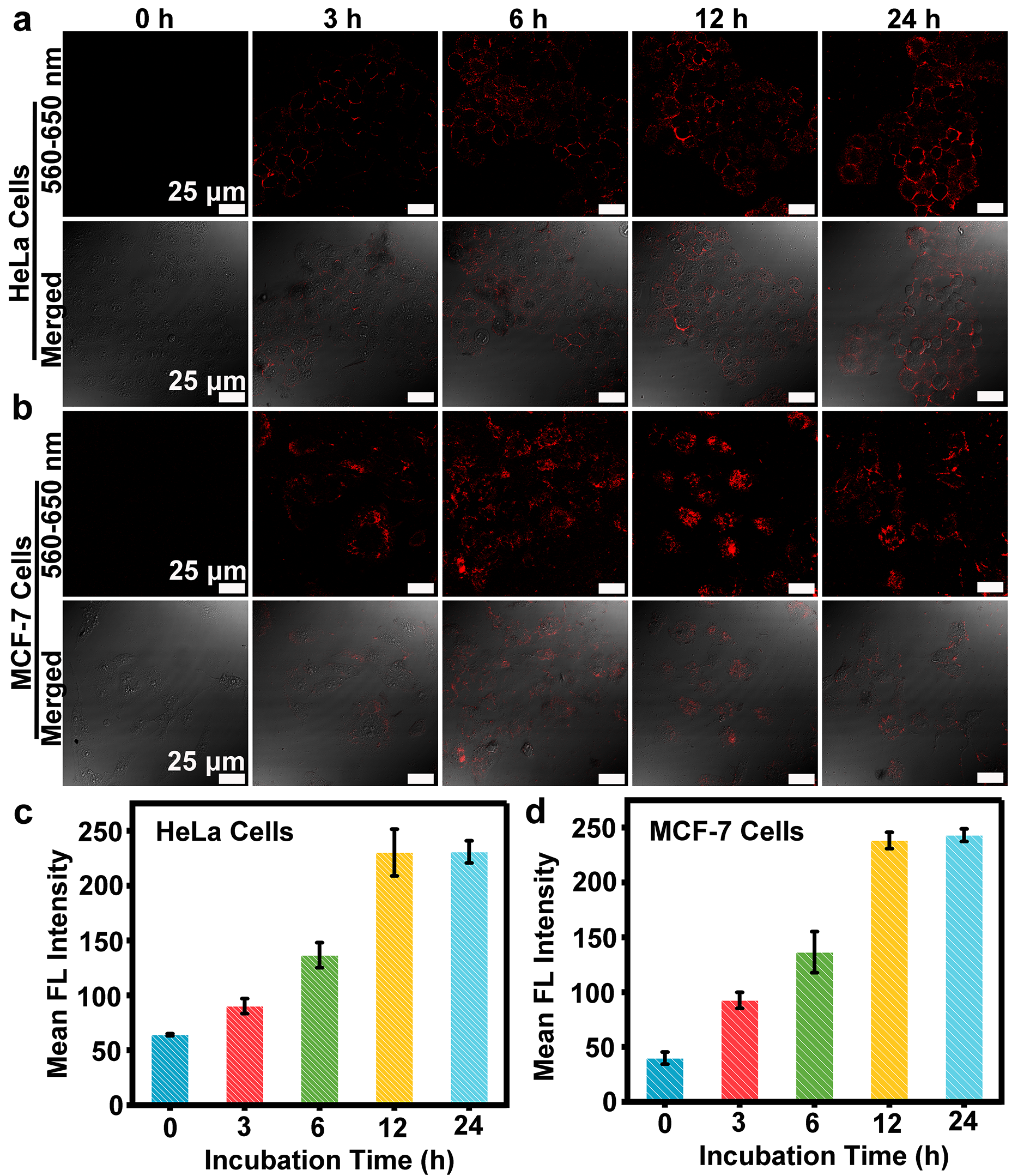


**Figure S13. Intracellular localization of TC/Apt with different incubation time**. (**a**) and (**b**) Confocal pictures of HeLa cells and MCF-7 cells treated by TC/Apt for different times (e.g., 0, 3, 6, 12, and 24 h) at 37 ^o^C. Scale bars, 25 μm. (**c**) and (**d**) Corresponding histograms of the fluorescence intensity of the TC/Apt-treated HeLa and MCF-7 cells after different times (e.g., 0, 3, 6, 12, and 24 h) at 37 ^o^C. All error bars represent the standard deviation obtained from three independent measurements.

As exhibited in **Figure S13**, as incubation time prolongs, red fluorescence signals are gradually enhanced in cellular cytoplasm, and reach the maximum intensity at 12 h. As thus, 12 h is selected as the optimal incubation time since fluorescence of the TC/Apt in live cells reaches the strongest intensity at this time.


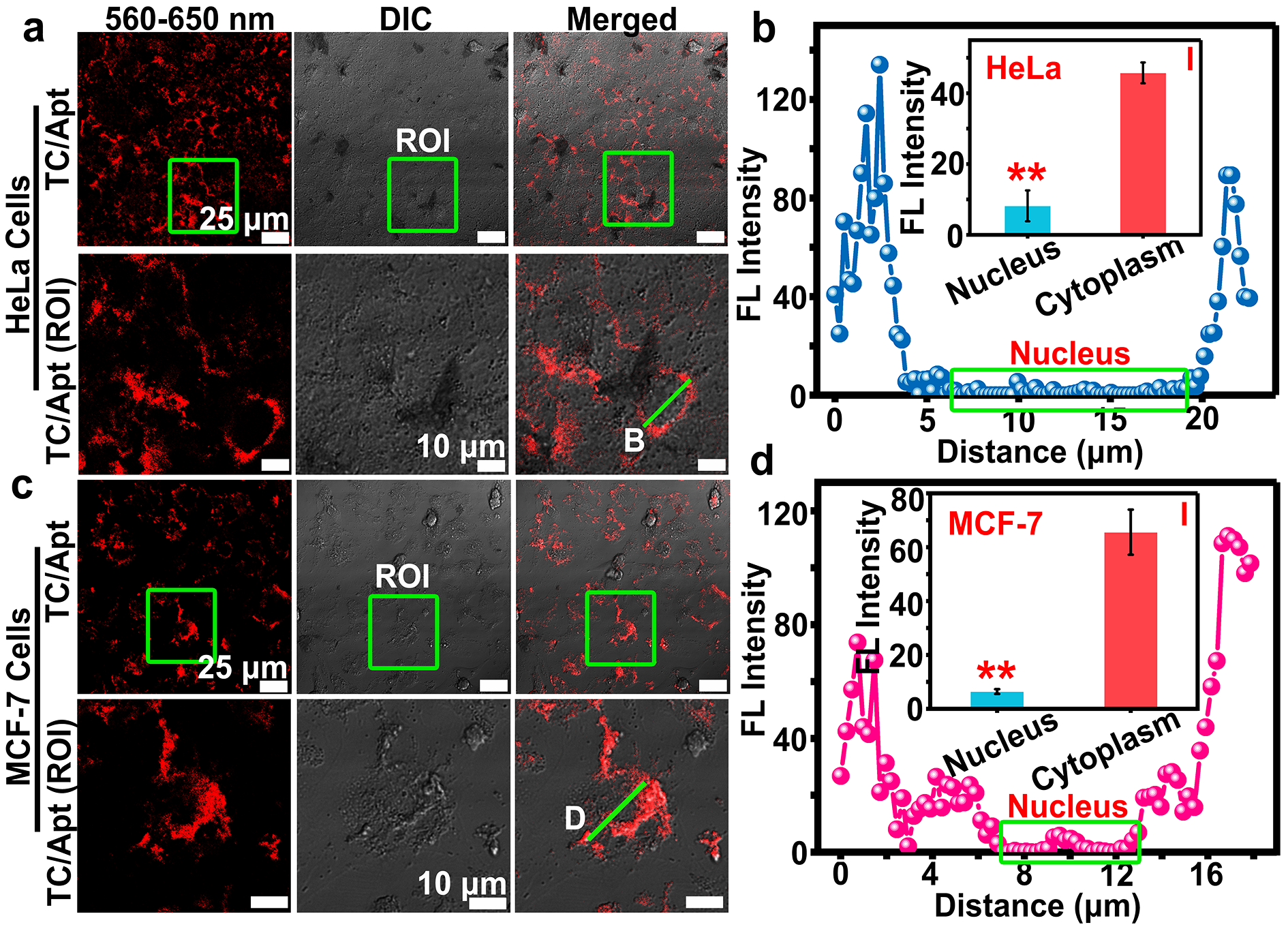


**Figure S14. Intracellular distribution of the TC/Apt.** Confocal images of TC/Apt in live HeLa cells (**a**) and MCF-7 cells (**c**). The cells are treated with 200 μg/mL TC/Apt at 37 ^o^C for 12 h. The distribution profiles of fluorescence intensity of TC/Apt along the diameter of a single HeLa cell (**b**) and MCF-7 cell (**d**). Insert I present corresponding histograms of mean fluorescence intensity in nucleus and cytoplasm. The error bars show the standard deviation determined from three independent measurements. ** represents p < 0.01.

As revealed in **Figure S14a** and **S14c**, red fluorescence signals are observed in cytoplasm rather than in nucleus for both HeLa and MCF-7 cells. In addition, as shown in region of interest (ROI) in **Figure S14b** and **S14d**, a green line crossing cytoplasm and nucleus generates the intensity profiles. Comparatively, the intensity profile of cytoplasm is significantly different from that of nucleus. Furthermore, as indicated in corresponding histograms of fluorescence intensity, the fluorescence intensity in cytoplasm is much higher than that in nucleus (e.g., ~5.6-fold enhancement of HeLa cells and ~10.3-fold enhancement of MCF-7 cells, p < 0.01).


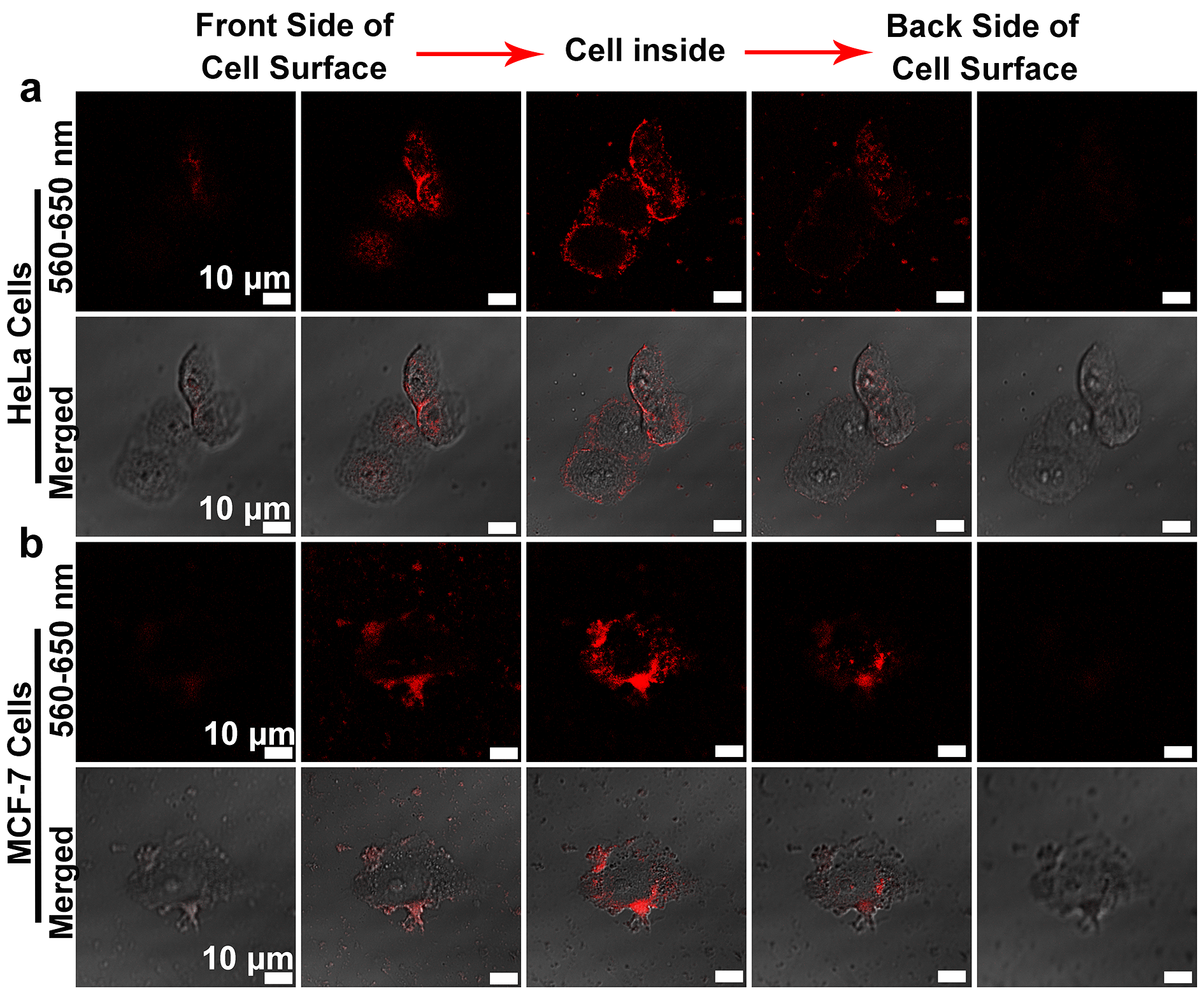


**Figure S15.** **Confocal images and reconstitution of HeLa and MCF-7 cells treated with the prepared TC/Apt.** (**a**) Confocal pictures of HeLa cells treated by the TC/Apt versus the changes of Z-axis. Scale bars, 10 μm. (**b**) Confocal pictures of MCF-7 cells treated by the TC/Apt versus the changes of Z-axis. Scale bars, 10 μm.

As presented in **Figure S15**, weak fluorescence is able to be detected on the front side of cell surface. As the scan proceeds towards the inner layer of HeLa or MCF-7 cells, the red fluorescence gradually increases. When the Z-axis changes to the cell inside, the strongest red fluorescence can be detected in cells. After that, the red fluorescence becomes weaker as the scan proceeds to the back side of cell surface. These results reveal that the TC/Apt are able to enter into cell and finally locate in cytoplasm, rather than simply adsorbing to the cell surface.

**8. Fluorescence cellular imaging of ATP in 4T1 cells with different treatments**


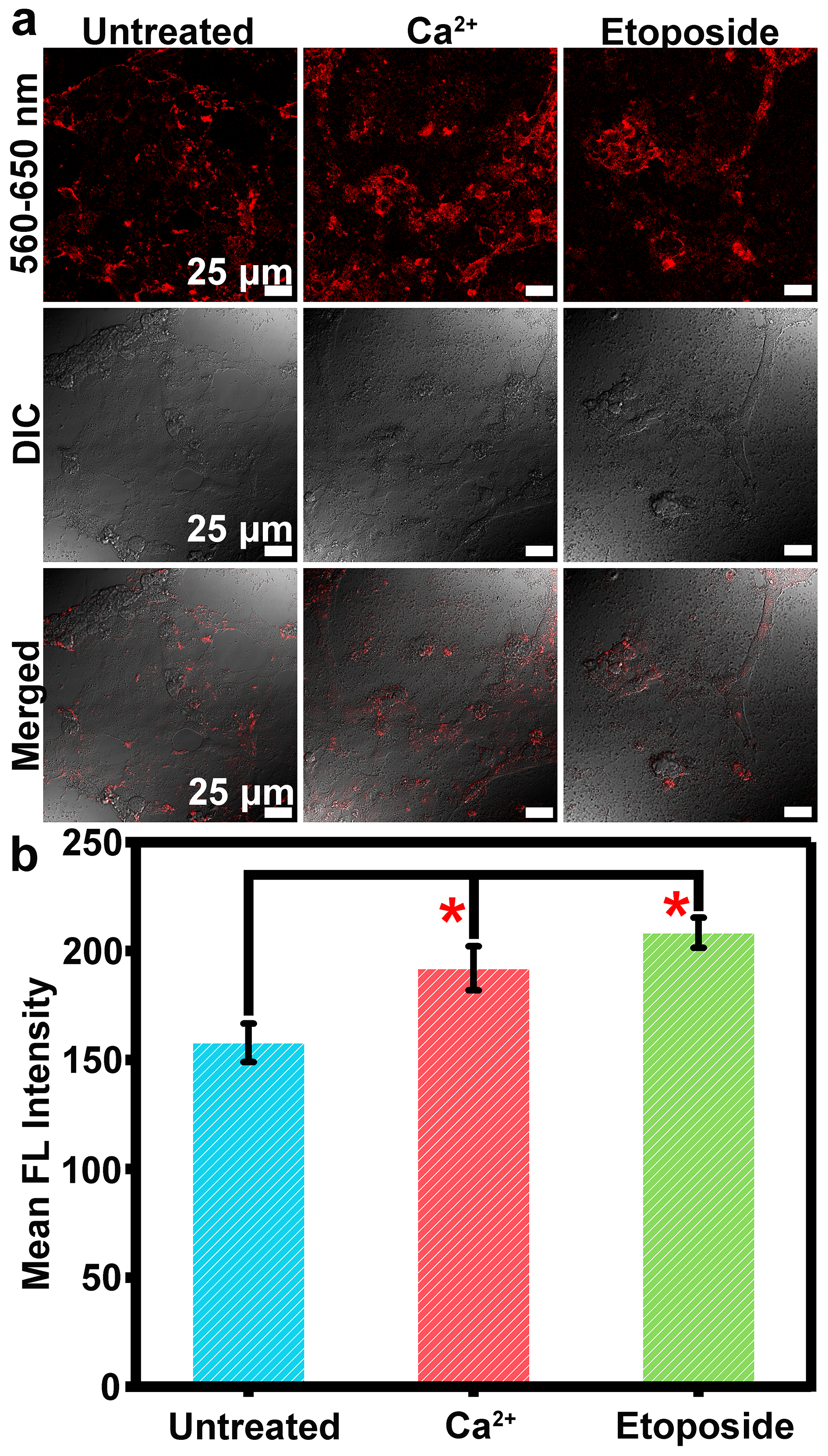


**Figure S16. Fluorescence cellular imaging of ATP in 4T1 cells with different treatments.** (**a**) Confocal images of TC/Apt-treated 4T1 cells further treated by pure medium (untreated), Ca^2+^ (5 mM), and etoposide (0.1 mM) for 2 h. Scale bars, 25 μm. (**b**) Corresponding histograms of fluorescence intensity of the TC/Apt-treated 4T1 cells further treated by pure medium (untreated), Ca^2+^ (5 mM), and etoposide (0.1 mM) for 2 h. All error bars represent the standard deviation obtained from three independent measurements.

As shown in **Figure S16**, for both 4T1 cells, in comparision to relatively weaker fluorescence of cells without incubation with Ca^2+^ or etoposide (untreated groups), relatively stronger fluorescence is measured in cells incubated with Ca^2+^ or etoposide (**Figure S16a**). For more semi-quantitative evaluation, mean fluorescence intensities of untreated, Ca^2+^ and etoposide groups are given in **Figure S16b**. In detail, compared with untreated cells, mean fluorescence intensity increases by ~17% in Ca^2+^-treated cells, by ~29% in etoposide-treated cells.

**9. References**

(1) Wang Y, Li Z, Hu D, Lin CT, Li J, Lin Y. Aptamer/Graphene Oxide Nanocomplex for in Situ Molecular Probing in Living Cells. J. Am. Chem. Soc. 2010; 132: 9274-6.

(2) Tan X, Chen T, Xiong X, Mao Y, Zhu G, Yasun E, Li C, Zhu Z, Tan W. Semiquantification of ATP in Live Cells Using Nonspecific Desorption of DNA from Graphene Oxide as the Internal Reference. Anal. Chem. 2012; 84: 8622-7.

(3) Liu Z, Chen S, Liu B, Wu J, Zhou Y, He L, Ding J, Liu J. Intracellular Detection of ATP Using an Aptamer Beacon Covalently Linked to Graphene Oxide Resisting Nonspecific Probe Displacement. Anal. Chem. 2014; 86: 12229-35.

(4) Yi M, Yang S, Peng Z, Liu C, Li J, Zhong W, Yang R, Tan W. Two-Photon Graphene Oxide/Aptamer Nanosensing Conjugate for In Vitro or In Vivo Molecular Probing. Anal. Chem. 2014; 86: 3548-54.

(5) Wen C, Huang Y, Tian J, Hu K, Pan L, Zhao S. A Novel Exonuclease III-aided Amplification Assay Based on A Graphene Platform for Sensitive Detection of Adenosine Triphosphate. Anal. Methods 2015; 7: 3708-13.

(6) He S, Qu L, Tan Y, Liu F, Wang Y, Zhang W, Cai Z, Mou L, Jiang Y. A Fluorescent Aptasensor with Product-Triggered Amplification by Exonuclease III Digestion for Highly Sensitive ATP Detection. Anal. Methods 2017; 9: 4837-42.

(7) Shamsipur M, Molaei K, Molaabasi F, Hosseinkhani S, Taherpour A, Sarparast M, Moosavifard SE, Barati A. Aptamer-Based Fluorescent Biosensing of Adenosine Triphosphate and Cytochrome c via Aggregation-Induced Emission Enhancement on Novel Label-Free DNA-Capped Silver Nanoclusters/Graphene Oxide Nanohybrids. ACS Appl. Mater. Interfaces 2019; 11: 46077-89.

(8) Ge J, Ou EC, Yu RQ, Chu X. A Novel Aptameric Nanobiosensor Based on The Self-assembled DNA-MoS_2_ Nanosheet Architecture for Biomolecule Detection. J. Mater. Chem. B 2014; 2: 625-8.

(9) Jia L, Ding L, Tian J, Bao L, Hu Y, Ju H, Yu JS. Aptamer Loaded MoS_2_ Nanoplates as Nanoprobes for Detection of Intracellular ATP and Controllable Photodynamic Therapy. Nanoscale 2015; 7: 15953-61.

(10) Fan YY, Mou ZL, Wang M, Li J, Zhang J, Dang FQ, Zhang ZQ. Chimeric Aptamers-Based and MoS_2_ Nanosheet-Enhanced Label-Free Fluorescence Polarization Strategy for Adenosine Triphosphate Detection. Anal. Chem. 2018; 90: 13708-13.

(11) Zhu X, Fan L, Wang S, Lei C, Huang Y, Nie Z, Yao S. Phospholipid-Tailored Titanium Carbide Nanosheets as a Novel Fluorescent Nanoprobe for Activity Assay and Imaging of Phospholipase D. Anal. Chem. 2018; 90: 6742-8.

(12) Mo R, Jiang T, DiSanto R, Tai W, Gu Z. ATP-triggered Anticancer Drug Delivery. Nat. Commun. 2014; 5: 3364.

(13) Shen Y, Tian Q, Sun Y, Xu JJ, Ye D, Chen HY. ATP-activatable Photosensitizer Enables Dual Fluorescence Imaging and Targeted Photodynamic Therapy of Tumor. Anal. Chem. 2017; 89: 13610-7.

(14) Zhou Y, Tozzi F, Chen J, Fan F, Xia L, Wang J, Gao G, Zhang A, Xia X, Brasher H, Widger W, Ellis LM, Weihua Z. Intracellular ATP Levels Are A Pivotal Determinant of Chemoresistance in Colon Cancer Cells. Cancer Res. 2012; 72: 304-14.

(15) Xuan J, Wang Z, Chen Y, Liang D, Cheng L, Yang X, Liu Z, Ma R, Sasaki T, Geng F. Organic-Base-Driven Intercalation and Delamination for the Production of Functionalized Titanium Carbide Nanosheets with Superior Photothermal Therapeutic Performance. Angew. Chem., Int. Ed. 2016; 128: 14789-94.

(16) Zhong Y, Peng F, Bao F, Wang S, Ji X, Yang L, Su Y, Lee ST, He Y. Large-Scale Aqueous Synthesis of Fluorescent and Biocompatible Silicon Nanoparticles and Their Use as Highly Photostable Biological Probes. J. Am. Chem. Soc. 2013; 135: 8350-6.

(17) Wang X, Yang Y, Zhou Y, Wu P, Chen H, Trefonas P. Hydrogen Bond Mediated Partially Miscible Poly (N-Acryloyl Piperidine)/ Poly (Acrylic Acid) Blend with One Glass Transition Temperature. Polymer 2018; 151: 269-78.

(18) Zhang Q, Wang F, Zhang H, Zhang Y, Liu M, Liu Y. Universal Ti_3_C_2_ MXenes Based Self-Standard Ratiometric Fluorescence Resonance Energy Transfer Platform for Highly Sensitive Detection of Exosomes. Anal. Chem. 2018; 90: 12737-44.

(19) Fotakis G, Timbrell J. A. In vitro Cytotoxicity Assays: Comparison of LDH, Neutral Red, MTT and Protein Assay in Hepatoma Cell Lines Following Exposure to Cadmium Chloride. Toxicol. Lett. 2006; 160: 171-7.

(20) Hu Q, Li W, Hu X, Hu Q, Shen J, Jin X, Zhou J, Tang G, Chu PK. Synergistic Treatment of Ovarian Cancer by Co-delivery of Survivin shRNA and Paclitaxel via Supramolecular Micellar Assembly. Biomaterials 2012; 33: 6580-91.
